# Recent trends in mesoporous carbon-based nanoplatforms for biomedical application

**DOI:** 10.1016/j.jpha.2025.101383

**Published:** 2025-07-03

**Authors:** Wei Yang, Jinnian Ge, Mohan Jiang, Nan Zhang, Qinghe Yang, Kaisheng Nan, Qinfu Zhao, Long Wan, Xiaofan Wang

**Affiliations:** a Department of Infectious Disease, Shengjing Hospital of China Medical University, Shenyang, 110004, China; b Department of General Surgery, The First Hospital of China Medical University, Shenyang, 110001, China; c Department of Cardiology, The First Hospital of China Medical University, Shenyang, 110001, China; d Department of Pharmacy, The First Hospital of China Medical University, Shenyang, 110001, China; e School of Pharmacy, China Medical University, Shenyang, 110122, China; f Department of Pharmaceutics, School of Pharmacy, Shenyang Pharmaceutical University, Shenyang, 110016, China; g Department of Pharmacy, The Fourth Affiliated Hospital of China Medical University, Shenyang, 110032, China

**Keywords:** Mesoporous carbon-based nanocomposites, Biomedical application, Tumor theranostics, Antibacterial, Biological detection

## Abstract

Mesoporous carbon nanoparticles (MCNs) have received considerable attention for biomedical applications due to their unique structural features, including high specific surface area, adjustable pore size, and remarkable biocompatibility. These properties have addressed key challenges such as inefficiencies in drug loading and release, minimizing the side effects associated with conventional treatments. In this review, the classification and the research progress of MCNs are summarized firstly, the preparation and modification techniques to enhance their functionality and properties are further reviewed, the main physicochemical properties are introduced as well, highlighting their contributions to MCNs in applications. In addition, the biomedical applications of MCNs are emphasized, including tumor therapy, tumor theranostics, antibacterial, delivery of active molecules and biological detection. Finally, the prospects and challenges of clinical application based on MCNs are analyzed to provide an effective reference and lay the foundation for further research.

## Highlights

• Classification, synthesis methods and modification strategies of MCNs were expounded.
• The physicochemical properties of MCNs and design strategies were highlighted.
• MCNs-based nanoplatforms for tumor therapy and tumor theranostics were presented.
• Antibacterial, biological detection and delivery of insoluble drugs were reviewed.
• The challenges, prospects and clinical future development of MCNs were prospected.

## Graphical abstract

**Figure:**
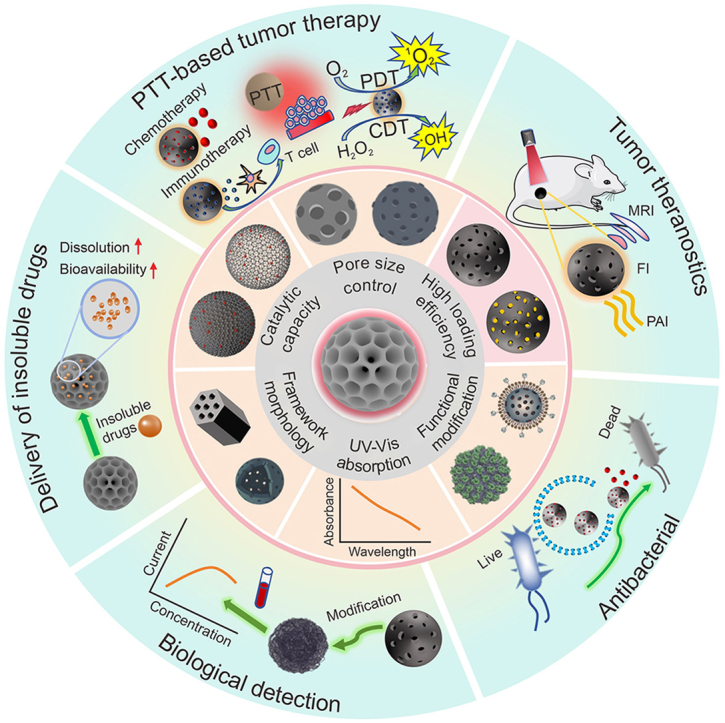

## 1 Introduction

Nanomaterials have attracted much attention in the domains of drug delivery, tumor therapy, bioimaging, antibacterial applications, and antioxidant research with the advances of nanotechnology [1]. In recent years, mesoporous nanomaterials, particularly mesoporous silica nanoparticles (MSNs), have shown substantial improvements and remarkable functionality [2]. These advances are attributed to their large surface area, modifiable surface, porous volume and adjustable pore size, which collectively improve abundant storage capacity and prevent premature release of guest molecules. Compared to sp^2^-based carbon nanocomposites [3] and conventional microporous nanomaterials, mesoporous carbon nanoparticles (MCNs) [4] have the features of mesoporous nanomaterials and carbon composition. Unlike metal-organic frameworks (MOFs), which often face challenges in structural stability under physiological conditions [5], MCNs exhibit superior chemical robustness and thermal stability, making them more suitable for biomedical applications requiring long-term durability. These features include: (1) large surface area and porous volume for high drug loading efficiency, (2) tailorable porous structure for precise drug release regulation, (3) supramolecular π-π packing for favourable loading of aromatic drugs with prolonged drug release performance, (4) easily functionalized surface for controlled and targeted drug delivery, (5) good biocompatibility, physical and chemical stability, and (6) distinctive optical characteristics for real-time tracking and bioimaging. Additionally, compared to MSNs and MOFs, MCNs performed outstanding performance in optical tunability and photothermal conversion efficiency, enabling enhanced multifunctionality in theranostic applications.

Furthermore, MCNs exhibit excellent light absorption, particularly in the near-infrared (NIR) band, demonstrating exceptional thermal conversion ability and photoacoustic imaging (PAI) performance. Photothermal therapy (PTT) has gained considerable popularity as an alternative for eradicating tumors [6,7]. PTT has exhibited prominent advantages, such as short-term treatment, low recurrence rate and high efficiency in the solid tumors treatment compared to conventional methods. Therefore, MCNs are extensively utilized in PTT-based cancer combination therapies, such as chemo-PTT, immunotherapy-PTT, photodynamic therapy (PDT)-PTT, and PTT-enhanced nanozyme-mediated catalytic therapy. PTT-induced local heating can reshape the physical and chemical properties of the tumor microenvironment (TME), facilitating rapid release of active ingredients from MCNs and enhancing their effect on tumor cells through increased molecular thermal mobility. Additionally, thanks to light illumination and hyperthermia, the chemical/enzymatic reactions can be facilitated, which is particularly favourable for nanodynamic and catalytic therapies [8]. Tumor-associated antigens exposed to T-cells after necrosis and apoptosis could trigger local antitumor responses, revitalizing the organism's anti-cancer immune response and facilitating targeted identification and elimination of metastatic cancer cells.

PTT-based synergistic therapy removed the limitations of thermal ablation in tumor treatment. Simple thermal ablation requires high temperature, so normal tissues are also damaged to some extent. In contrast, PTT leverages photothermal transduction agents (PTAs) to locally heat tumors, minimizing side effects. Among the many PTAs, carbon materials can overcome the disadvantages of high cost and non-degradation of PTAs of noble metal materials [9]. Among the many PTAs based on carbon materials, MCNs have a wide absorption in the wavelength range of 650–900 nm and are very suitable for PTT and PAI. Meanwhile, MCNs are more suitable for PTT due to their excellent drug loading capacity and easy surface functionalization [10]. MCNs can be modified to make them clustered in the tumor site and obtain PTT effect. In addition, MCNs can be loaded with chemotherapeutic drugs or photosensitizers to realize PTT-based synergistic chemotherapy or PDT [11]. This capability makes it possible for the application of MCNs for PAI-guided PTT in malignancies, facilitating both detection and treatment.

Nanozymes are catalytic nanomaterials that exhibit enzyme-like behaviours [12]. Unlike natural enzymes, nanozymes are simple to create, reusable and may be functionally modified [13]. MCNs demonstrate superior enzyme-like capabilities in comparison to other mesoporous substances. Generally speaking, the graphite structure formed by MCNs during the high-temperature carbonization process is capable of converting oxygen into different oxides and water through oxygen reduction reactions, facilitating the production through oxygen evolution reactions under extreme conditions [14]. MCNs are reported to possess peroxidase (POD)-like enzyme activity which can catalyze hydrogen peroxide (H_2_O_2_) to generate hydroxyl radicals (·OH) in the TME, the most powerful oxidizing free radicals. MCNs are reported to have glutathione (GSH) oxidase activity, which is obviously higher than that of graphene oxide, a carbon family material [15].

Furthermore, the variety of enzyme activities of multiplexed MCN can be enhanced through the incorporation of nitrogen components or metallic components (copper ion [16,17], iron ion [18], and manganese [19]) into the mesopores framework, facilitating the biomedical application of MCNs. Nanozymes with catalase or SODase characteristics can produce oxygen to tumor sites, enabling effective implementation of nanodynamic therapies depending on oxygen. Nanozymes can partially substitute natural enzymes in the combined treatment of cancer. Upon illumination, the surface of MCNs becomes energized, accelerating enzyme-like reactions such as peroxidases/oxidases. This process not only directly annihilates tumors through the emission of massive quantities of reactive oxygen species (ROS), but also undermines the equilibrium of redox, which renders tumors more vulnerable to anticancer drugs and ROS damage. Beyond tumor theranostics, carbon-based nanozymes play crucial roles in other fields due to diversity of catalytic mechanisms and catalysis. Nanozymes exhibit antibacterial characteristics, making them suitable for treating bacterial infections [20]; Nanozymes can be served as broad-spectrum antioxidants in managing inflammation [21] and can be examined via traditional colorimetric techniques and electrochemistry [22].

**Scheme 1:**
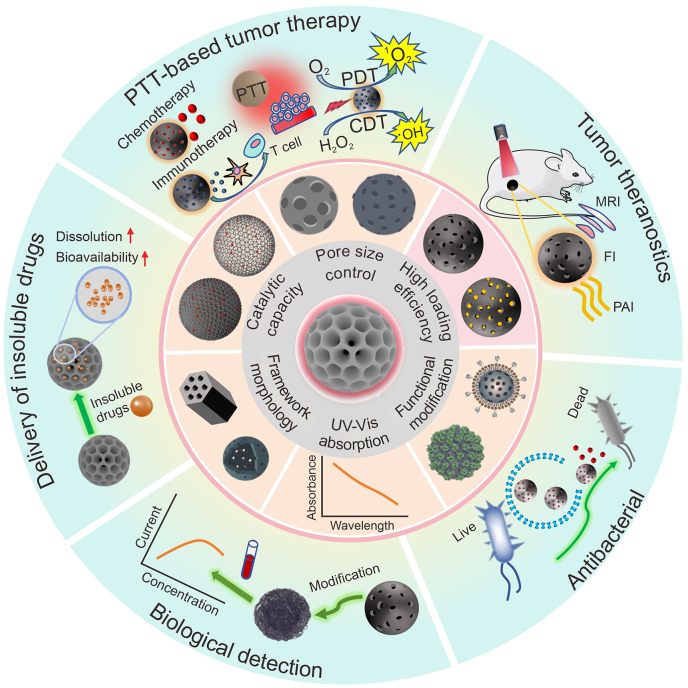
The physicochemical properties and biomedical applications of mesoporous carbon-based nanoplatforms. PTT: photothermal therapy; PDT: photodynamic therapy; CDT: chemodynamic therapy; MRI: magnetic resonance imaging; FI: fluorescence imaging; PAI, photoacoustic imaging; UV-Vis: ultraviolet-visible.

MCNs-based synthesis strategies, modification strategies and biocompatibility [23], photothermal-enhanced synergistic therapy [24], and drug delivery [25] have been reviewed previously. In recent years, nanoplatforms based on MCNs have made significant strides in low-power and efficient PTT-based theranostics, carbon nanozymes, degradable composite carbon carriers [26], *in vivo* diagnosis and imaging [27]. However, reviews focusing on the biomedical applications of MCNs, alongside these recent research advancements, have not been conducted. In this review, we primarily summarize recent progress in the drug delivery aspects of MCNs, including (1) classification of MCNs, (2) preparation and modification of MCNs, (3) fundamental physicochemical properties of MCNs, and (4) applications of mesoporous carbon-based nanoplatforms (Scheme 1). In addition to this, we also emphasize the present biomedical uses of mesoporous carbon nanomaterials, such as PTT, delivering biotherapeutic agents, and assisting in bioimaging. This review also outlines the challenges and prospects for MCNs. We believe that via meticulous design, precise preparation, and methodical investigation, MCNs will progressively be integrated into clinical applications, playing an invaluable function in field of biomedical science.

## 2 Classification

**Scheme 2:**
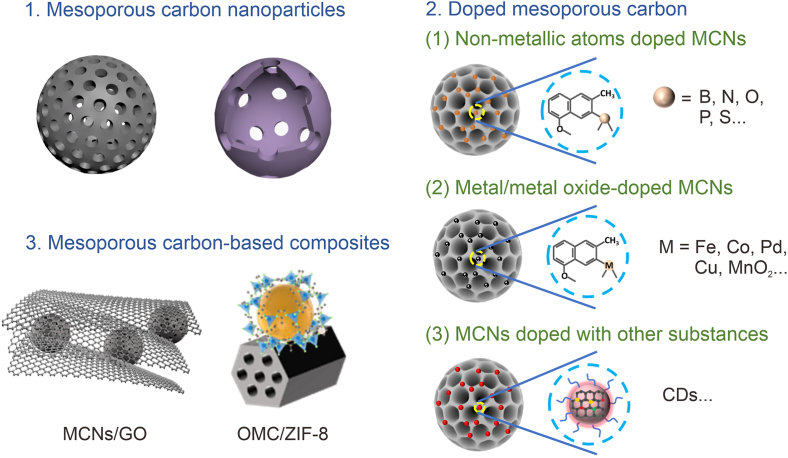
The morphological feature classification of MCNs. MCNs: mesoporous carbon nanoparticles; CDs: carbon dots; GO: graphene oxide; OMC: ordered mesoporous carbon; ZIF-8: zeolitic imidazolate frameworks-8.

In recent years, mesoporous carbon-based nanoplatforms have found widespread applications in biomedicine. Given these inherent physicochemical properties, a wide variety of mesoporous carbon-based nanoplatforms have been developed to date. Basically, mesoporous carbon-based nanoplatforms can be divided into three categories, mainly including MCNs, doped mesoporous carbon and mesoporous carbon-based composites (Scheme 2). These nanoplatforms with different structures have their unique advantages in biomedical application.

### 2.1 MCNs

The MCNs refer to carbon materials featuring a well-defined porous structure with pore diameters ranging between 2 and 50 nm. MCNs exhibit distinctive characteristics including high specific surface area, tunable pore size distribution, excellent chemical stability, and hierarchical pore architecture. The unique physicochemical properties of MCNs, such as their exceptional electrical conductivity, thermal stability, and surface modifiability, make them particularly suitable for applications in energy storage, catalysis, and adsorption technologies. In contrast to carbon nanotubes (which pose potential risks of cellular membrane penetration) and graphene (whose sharp edges may induce membrane damage), the spherical or bulk morphology of MCNs demonstrates superior biocompatibility with reduced cytotoxicity, making MCNs particularly advantageous for biomedical applications. This combination of structural controllability, surface functionality, and biological safety establishes MCNs as versatile nanoplatforms in nanomedicine. Carbon-based mesoporous materials usually possess better pore volume and specific surface area, which is conducive to drug delivery. Compared with mesoporous materials represented by MSNs, the carbonization process endows MCNs with higher surface area (typically 500–1500 m^2^/g) and larger pore volume (0.5–2.5 cm^3^/g). Notably, aromatic groups derived from carbon precursors remain on the MCNs surface during pyrolysis. The π-π conjugation and supramolecular stacking interactions between these aromatic groups and drug molecules significantly enhance drug loading capacity.

In particular, the use of hollow MCNs (HMCNs) can achieve a high drug loading capacity of 80% for doxorubicin (DOX) [28]. When active agents (chemical agents, photosensitizers, and contrast agents) are loaded into the porous carbon skeleton, the rigid shell of MCNs can protect them from environmental influences. For example, the photosensitizer chlorin e6 (Ce6) can be rapidly inactivated under weak light conditions (0.5 W/cm^2^). However, due to the protective effect of MCNs, Ce6-loaded MCNs can maintain uniform stability in sunlight for 15 days [29]. The abundant surface-active sites formed by carbonization also provide a carbon matrix for surface modification. On the one hand, macromolecular modification, phospholipid coating, and biofilm coating on the surface of MCNs provide MCNs with excellent biocompatibility and long circulation performance. On the other hand, antibody modification and clever charge responsiveness flipping also provide broad research ideas for selective organelle targeting and improving the enrichment efficiency of lesion sites.

MCNs have strong light absorption ability at 300–1400 nm. When MCNs absorb light, electrons transition from the ground state to the excited state, relax as the energy decays through non-radiative decay, and the surrounding environment becomes overheated due to the increase in kinetic energy. On the one hand, this generated thermal expansion can propagate outward and be converted into ultrasonic signals, giving MCNs the ability of PAI. On the other hand, the thermal effect can change the physiological environment of the treatment site to achieve a better therapeutic effect, including the following aspects: (1) The thermal effect intensifies the molecular motion of MCNs, which makes the electron transfer on the surface of MCNs more active, manifesting higher enzyme-like catalytic activity [30]. (2) Photothermal promotes the breakage of surface-modified thermosensitive bonds and the accelerated release of loaded drugs. (3) Mild thermal effects promote local blood flow and immune effects, relieve the lack of oxygen. in tumor sites. (4) The intense photothermal effect induces local apoptosis and necrosis through the caspase-3-mitochondria axis, leading to vascular closure, local edema and cell necrosis [31]. (5) Thermal signals regulate protein synthesis and cell cycle, such as upregulation of the heat shock proteins (HSPs) family [32]. Based on the above photothermal properties, a series of photothermal combined therapies based on MCNs have been proposed. The drug loading capacity increased through the drug loading characteristics of MCNs themselves, avoiding the phototoxicity and photodegradation of photosensitive drugs. MCNs regulate cell signaling pathways and remodel the surrounding physiological environment through photothermal effect to enhance the therapeutic effects of MCNs in anti-tumor, anti-inflammatory, antibacterial and other fields.

### 2.2 Doped mesoporous carbon

#### 2.2.1 Non-metallic atoms doped MCNs

During high-temperature processing, the introduction of trace oxygen and nitrogen elements forms functional groups (e.g., amino, carboxyl, and hydroxyl) that significantly alter the hydrophilicity/hydrophobicity of MCNs. These surface modifications enhance MCNs' adsorption capacity for reactants, thereby increasing substrate concentration on their surfaces. Simultaneously, the graphitic structure generated from carbon precursors promotes efficient electron transfer, endowing MCNs with versatile catalytic activities. Notably, increasing non-metallic dopant density (such as N, B, and P) elevates electron density and catalytic performance. Liang et al. [33] exemplified this principle by engineering nanoparticles (NPs) with tunable enzyme-like activities through controlled N/B/P doping, ultimately selecting optimal POD-like NPs for antitumor applications. Structural optimization extends beyond catalytic enhancement. Phosphorus doping introduces P–O groups that address MCNs' inherent hydrophobicity limitation, improving both hydrophilicity and drug-loading efficiency. Moreover, phosphorus modifies physical parameters including specific surface area, pore size distribution, and pore volume, critical factors influencing drug delivery dynamics [23].

The synergy between heteroatoms creates properties unattainable through single-element doping. P/N co-doping exemplifies this phenomenon: nitrogen atoms adjust chemical properties while phosphorus introduces functional groups, synergistically enhancing conductivity and creating additional binding sites for drug delivery applications [34]. Similarly, N/B co-doping generates complementary acid-base sites and enriched electronic states. Zhu et al. [35] demonstrated this through nitrogen-boron codoped yolk-shell nanospheres, where enhanced conductivity and surface polarity enabled superior simultaneous detection of xanthine and guanosine with remarkable durability. The structural advantages of such systems facilitate electron transport and biomolecule penetration. Beyond biomedical applications, heteroatom combinations like N/S, N/O, and P/O co-doping have found broader utility in energy and environmental fields, demonstrating the versatility of heteroatom-engineered MCNs.

#### 2.2.2 Metal/metal oxide-doped MCNs

Metal ions exhibit significant application potential in biomedical fields due to their unique coordination chemistry and optoelectromagnetic properties, including fluorescence emission and paramagnetism. Conventional metal-based agents frequently undergo systemic distribution *in vivo*, leading to toxic accumulation in non-target organs. The π-coordination interactions and chelation effects between metal ions and organic carbon sources (e.g., phenolic resins) effectively reduce systemic toxicity while enhancing the photothermal conversion efficiency of MCNs and conferring multimodal imaging capabilities.

The strong metal-carbon interactions optimize metal dispersion. Through spatial confinement or coordination regulation, single-atom dispersion can be achieved to prevent aggregation-induced deactivation, suppress nanoparticle coalescence, and enhance electron transport. Su et al. [36] constructed nitrogen-doped MCNs nanozymes with single atom iron dispersion for enhancing PTT. The single atom Fe in nanozymes exhibited CAT-like and POD-like enzyme activity in TME, and the separated N atom forms a stable Fe–N coordination structure with Fe atom, providing abundant active sites. To further reduce reaction energy barriers in catalytic processes, bimetallic systems synergistically activate reactants through coupling with carbon's sp^2^ hybrid orbitals, significantly improving hydrogen evolution reaction efficiency. Typical examples include Cu–Fe co-doped mesoporous carbon as enzyme-like materials for biomedical applications [18] and iron-zinc oxide-doped mesoporous carbon as porous drug delivery systems for TME [37].

Beyond structural engineering, metal atom doping could inject electrons or holes into the carbon matrix, modulating the material's Fermi level position, optimizing adsorption energy of reaction intermediates, and reducing activation energy. Furthermore, certain metal nanoparticles (e.g., Pt, Pd, and Cu) or single atoms (e.g., Fe, Co single-atoms) directly serve as catalytic active centers by participating in electron transfer processes during redox reactions. For example, Feng et al. [38] constructed copper doped HMCN for PDT of tumors. Doping Cu^2+^ significantly increased the POD-like activity and GSH depletion ability of the nanoparticles, enhancing the sensitivity of TME to PDT. The hypoxic state and antioxidant system of TME severely limit the efficacy of PDT [39]. Li et al. [40] doped MnO_2_ into MCN to enhance its PDT effect while giving the system dual-mode imaging capability (PAI/magnetic resonance imaging (MRI)). MnO_2_ can catalyze the production of hydrogen peroxide to generate O_2_ and utilize GSH-OXD activity to disrupt the antioxidant system of tumors.

#### 2.2.3 Mesoporous carbon doped with other substances

In practical applications, it is efficient to combine metal/metal oxide, non-metal, nanoparticles and other doping approaches to realize mesoporous carbon-based carriers for different design purposes. For instance, tight carbon-carbon bonds or metal-carbon bonds are unfavorable for the outer layer hydration and subsequent degradation of the carriers, which may lead to potential bioaccumulation toxicity. The combination of MCNs with Si can not only improve the stability and biocompatibility of the material, but also significantly accelerate the degradation rate of the particles. This doping strategy aims to achieve higher control and efficacy of drug release and bioimaging, thereby providing more research possibilities for targeted therapy and real-time imaging techniques [26]. The modifiable nature of the MCNs surface has a variety of derivatives. The combination of MCNs and carbon dots (CDs) has become a new *in vivo* real-time imaging technology. As a fluorescent probe, CDs can visualize the drug administration process, expanding the range of application. In addition, the cell marker fluorescence is photostable and performs better than organic fluorescent dyes. As for drug delivery, the disulfide bond between CDs and MCN@Si can be broken at low pH and high GSH concentration, which is beneficial for controlling drug release [41].

### 2.3 Mesoporous carbon-based composites

The above materials are mainly carbon structures, which have limitations in spite of the modification. When the proportion of other elements gradually increased and the carbon content reduced, some problems can be solved. Carbon-silica nanocomposites (CSNs) have solved the degradation problem of traditional materials well. Compared with traditional materials that need to be degraded by enzyme-catalyzed reactions under relatively strict conditions, CSNs can be degraded into small particles of 5 nm in simulated body fluids and lysozyme solutions without the assistance of enzymes within 16 days. This material will also serve as an immune adjuvant to produce tumor-associated antigens and mature dendritic cells [42]. When combined with NIR light, CSNs can induce the production of tumor-associated antigens, further expanding its application prospects in tumor treatment. In addition, metal elements were selected to synthesize different composite materials. Mesoporous carbon-titanium dioxide nanocomposites presented an ordered hexagonal mesocrystalline phase and showed good photocatalytic activity for the photodegradation of rhodamine B, which was beneficial for fluorescent cell staining [43]. Similarly, mesoporous carbon hollow spheres functionalized with manganese dioxide, which had good electrocatalytic activity and had been successfully used to detect the content of alpha-fetoprotein in human serum, making great contributions to the early diagnosis and prognosis of liver cancer [44].

The combination with other elements can not only optimize the performance of MCNs, but also expand the application of MCNs in sensing detection. Transition metal-doped carbon materials are promising platforms for glucose detection. Glucose sensors made of nickel NPs embedded in nanoporous carbon nanorods (Ni/MCNs) can detect glucose concentration in human blood samples in a short time due to their excellent stability and anti-interference [45]. Metal-doped composites also showed great potential in tumor therapy. As a representative example, carbohydrate antigen 19–9 (CA19-9) is a carbohydrate antigen and protein that is highly associated with malignant tumors. CeO_2_/FeOx@mC embedded in mesoporous carbon (CeO_2_/FeOx@mC) could sensitively detect CA19-9 as a cancer marker, which was of great significance for the treatment of pancreatic cancer, gastric cancer, colorectal cancer, among others [46].

## 3 Preparation and modification

### 3.1 Preparation

Due to the wide application of MCNs and their derivatives in the medical field, they have received extensive attention in recent years and an increasing number of preparation methods of MCNs have emerged. High-temperature carbonization is currently the main method for preparing MCNs. In this process, carbon precursors undergo carbonization on the surfaces of soft or hard templates under high-temperature conditions, and dense mesoporous carbon carriers are obtained after template removal. However, this high-temperature approach also introduces challenges such as strong hydrophobicity and a tendency for MCNs to aggregate in aqueous environments. In addition to our previous discussion on improving MCNs' surface properties through elemental doping (e.g., N and P) in the doping section, this section focuses on enhancing the hydrophilicity and biocompatibility of MCNs via surface modification approaches.

#### 3.1.1 Template strategies

Given that MCNs prepared by template strategies offer a series of attributes such as adjustable pore structure, narrow aperture distribution, orderly channel arrangement, large specific surface area and internal interconnecting pore network, template strategies are currently acknowledged as one of the most well-established techniques for MCNs construction. Generally, based on the differences in structure and property, template methods are segmented into hard template methods, soft template methods and multi-template methods [47].

##### 3.1.1.1 Hard template methods

Hard template methods principally rely on the growth of precursors in the nanoscale pores of the pre-prepared rigid template. Hard template methods for manufacturing MCNs generally possess the following procedures: (1) select the appropriate template material, which is equipped with specific composition and pore structure (anodized aluminum, zeolite molecular sieves, mesoporous materials, colloidal crystals and carbon nanotubes, etc*.*); and then (2) fill the pores of the template with carbon precursors; (3) carbonize the composites under the protection of inert gas; (4) add acid or alkali substances to etch the template to obtain MCNs [48]. For example, classic SBA-15 ordered silica template was used, filled with phenolic resin as carbon precursor and carbonized in nitrogen/argon to obtain a carbon-silica composite. The SBA-15 hard template was etched with hydrofluoric acid or sodium hydroxide to obtain a carbon support with ordered pores.

##### 3.1.1.2 Soft template methods

*Via* Soft template methods Via soft template methods, MCNs can be synthesized by adjusting the reaction temperature, time, and solvent types. Unlike hard template methods, which have considerable limitations such as requiring physical templates like polystyrene balls or SiO_2_ balls, soft template methods offer several advantages. Compared to hard template methods, soft template methods have larger specific surface area, and simultaneously, they can also synthesize mesoporous carbon of different morphologies. The main preparation process includes (1) self-assembly of carbon source (mainly phenolic resin) with surfactant as template to prepare ordered structure, (2) removal of surfactant, and (3) carbonization of mesoporous polymer to obtain continuous carbon framework. Several methods for synthesize mesoporous carbon materials by soft template methods include (1) evaporation induced self-assembly method, (2) water phase synthesis method, (3) macroscopic phase separation method, and (4) hydrothermal synthesis method. These approaches have their respective strengths and weaknesses.

Although the soft template methods are simple, there are also some shortcomings: (1) Monodispersity is difficult to control; (2) scale production is limited. Given the previously mentioned agglomeration and its costly preparation, it is not easy to get high-quality, scale-able MCNs; (3) The ore structure and size are not easily controlled.

#### 3.1.2 Monomicelle assembly method

The MCNs prepared by single micelle assembly have the characteristics of large specific surface area, long cycle life, high sodium ion storage capacity and rich nitrogen content. For example, Peng et al. [49] developed a programmable shear-induced dynamic assembly method to synthesize MCNs with a radial gradient structure. By adjusting the shear force, the micelle structure could be efficiently tuned. More importantly, by building on-demand agitation models, the synthesis process could be performed in a programmable way to enable self-assembly. Through single-micelle assembly strategies, diverse functional architectures have been synthesized including Janus nanocomposites, monolayered mesoporous nanosheets, mesoporous single-crystal nanoparticles, and micelle-derived porous liquids [50].

Otherwise, for the purpose of enhancing the series properties such as hydrophobicity, surface wettability, adsorption and stability of MCNs, some non-metallic elements (N, P, S, B) and mental elements (Fe, Co, Ni, Cu, Ti) can be doped to introduce the catalytic active site to improve the hydrogenation modification and oxidation activity, which is capable of improving the structure of MCNs, thus giving the modified MCNs unique properties.

### 3.2 Functional modification

With the continuous exploration of the application of MCNs and their derivatives, mesoporous carbon synthesis methods have gradually evolved. Because of the lack of functional groups or active sites on the surface of the pore walls, the application of pure mesoporous carbon materials is limited to some extent. To expand the application of mesoporous carbon, surface oxidized MCNs, targeting MCNs, and stimulus-responsive MCNs were obtained through functional modification.

#### 3.2.1 Surface oxidation

Due to the carbonaceous framework of MCNs generated by calcination or hydrothermal treatment at a high temperature, the nature of MCNs is highly hydrophobic. The introduction of oxygen-containing functional groups such as hydroxyl and carboxyl groups can effectively enhance the hydrophilicity and dispersibility of MCNs, while facilitating subsequent functional modifications. Researchers oxidize MCNs with strong acids to enhance hydrophilicity. Strong acid can lead to oxidation of the mesoporous carbon surface and the formation of hydrophilic functional groups such as carboxyl, hydroxyl and phenolic hydroxyl groups. The addition of these functional groups would significantly improve the hydrophilicity of MCNs. For example, Han et al. [51] introduced a large number of carboxyl groups (−COOH) on the surface of MCNs by oxidizing MCNs with a mixed solution of H_2_SO_4_ and H_2_O_2_, increasing the polarity of the carrier and avoiding the self-aggregation and adsorption of plasma proteins of the original MCNs in a polar environment. The carvedilol-loaded MCNs were injected into mice at a dose of 100 mg/kg. No abnormal behavior or death was observed in mice during subsequent stimulation experiments. As one of the simplest and most convenient carbon surface modification methods, surface modification can effectively improve the surface hydrophobic by attaching oxygen-containing groups.

#### 3.2.2 Targeting modification

Introducing specific ligands or antibodies onto the surface of MCNs can achieve specific recognition and targeting of targeted cells by MCNs. This design enables drugs to reach the lesion area more accurately, reducing damage to healthy tissues. Tumor cells often exhibit characteristics different from normal cells due to genetic instability. For example, interleukin-6 (IL-6) receptor is significantly overexpressed in ovarian cancer cells, but less expressed in normal tissues. Therefore, Wang et al. [52] chose I6P8 peptide as the targeting part of IL-6 receptor and constructed a therapeutic platform consisting of I6P8 peptide and DOX loaded oxidative MCNs. The results of cell uptake and tumor sphere penetration experiments showed that the binding of I6P8 peptide to oxidized MCNs significantly enhanced the accumulation and penetration of IL-6 receptor-mediated DOX in tumor spheres. The more specific target of the drug carrier, the better the overall therapeutic effect. The target can not only be cells, but also subcellular structures. Wang et al. [53] modified MCNs with folic acid (FA) and peptide M27-39 for the treatment of colorectal tumors. Due to the increased number and activity of FA receptors in tumor cells, FA enhances drug accumulation in colorectal tumor tissues, while the small peptide M27-39 can target and interfere with the metabolism of colorectal tumor mitochondria. In summary, targeted modified MCNs can enhance the effectiveness of drug therapy, increase bioavailability, and reduce systemic side effects.

#### 3.2.3 Stimulus-responsive release

As drug carriers, MCNs have a certain responsiveness to external stimulus. For example, the slightly acidic conditions of TME can weaken the connection between MCNs and certain drugs connected through hydrophobic interactions and π-π stacking [10]. However, the response of MCNs to external stimuli faces the challenges of being imprecise and not broad enough. Therefore, it is necessary to modify MCNs with stimulus-responsive molecules to improve and expand the application of MCNs-based drug delivery systems. The stimulus-responsive MCNs-based drug delivery systems reduce the loading or blocking effect of modified structures on drugs at the target site through conditional stimulation, thereby promoting drug release. The stimulation conditions are diverse, commonly including pH, temperature, laser, GSH, etc. Gisbert-Garzarán et al. [54] constructed a type of DOX-loaded MCNs released only a small amount of drug at physiological pH, while a large amount of drug was released at the slightly acidic pH of TME, thereby achieving significant cell growth inhibition on human osteosarcoma cells. Of course, more intelligent drug delivery systems with multiple responses can also be designed to improve the accuracy of drug release. Feng et al. [55] developed a HMCN platform that responded to triple stimulation of GSH, pH, and NIR laser to release DOX for the combination therapy of chemotherapy and PTT, with a cell apoptosis rate of over 95% in the combination therapy group. Nowadays, researchers tend to combine targeting ability with stimulus-responsive function. Fang et al. [56] developed a HMCN modified with hyaluronic acid (HA) and graphene quantum dots. The modification of HA endowed the nanoplatform with targeting for cancer cells. In addition, the nanoplatform also responded to dual stimuli of NIR laser and pH. As an important development direction of modern drug delivery systems, stimulus-responsive drug delivery systems, similar to targeted drug delivery systems, can also increase drug accumulation in lesions and reduce side effects.

## 4 Fundamental physicochemical properties

MCNs-based nanoplatforms have unique physicochemical properties that make them ideal for biomedical applications. Their structure allows for significant versatility in drug delivery systems, imaging, and therapeutic interventions. This section discusses these fundamental properties, highlighting their contribution to the effectiveness of MCNs in biomedical applications.

### 4.1 Large surface area and controllable pore size

Mesoporous carbon-based nanoplatforms are characterized by their large surface area and controllable pore size, contributing to various biomedical applications. The high surface area of MCNs allows them to have more active sites, which can increase the probability of substrate collision with the enzyme and improve the efficiency of the nanozymes. Sang et al. [57] designed Fe^3+^-MCN and proved that its HRP-like enzyme activity was higher than that of Fe_3_O_4_ NPs with the same iron content. The pore size of MCNs can be finely regulated during the synthesis process, allowing for precise control over their structural properties and functionality. One of the most effective methods is through the calcination of MCNs, which involves heating the materials at high temperatures to promote the growth and enlargement of pore structures. Chen et al. [58] prepared PLMC (a mesoporous carbon derivative material with a pore size of 4.8–16.2 nm) by calcining PLNP@UIO-66 (a composite material with pore size 1.1 nm), which greatly expanded the application range and drug loading capacity of the material. For some macromolecular drugs, having larger pore sizes is particularly beneficial as it facilitates the stacking of the drug molecules within the pores, thereby optimizing drug adsorption and improving overall loading capacity. Ghosh et al. [59] proved that MCNs with larger pore size showed superior adsorption and slowed down release effects on macromolecule drugs, which proved to be efficient carriers of macromolecule drugs. Undoubtedly, large surface area and controllable pore size provide a broad platform for mesoporous carbon delivery of various drugs.

### 4.2 Framework and morphology control

With large specific surface areas and tunable mesopores, MCNs are served as effective heteroatom doping carriers for constructing multifunctional nanoplatforms. By introducing specific functional components, MCNs with diverse functionalities have been designed. For example, incorporating contrast agents into the MCNs framework can endow MCNs with imaging capabilities, demonstrating their potential for theranostic applications. Zhang et al. [60] prepared ordered mesoporous carbons (OMCs) doped with highly dispersed γ-Fe_2_O_3_ and GdPO_4_ nanoparticles using a one pot in-situ method. The nanoplatform exhibited MRI imaging effects, while the highly dispersed doping ensured the mass transfer efficiency and stability of the nanoplatform. Framework regulation can achieve the degradation of mesoporous carbon carriers and prevent their accumulation *in vivo* to improve biocompatibility. Lv et al. [27] developed a carrier composed of Co_3_S_4_ and nitrogen doped mesoporous carbon for PTT in tumors, which can fragment into sub-5 nm nanoparticles under mild acidity. Framework regulation can also prevent drugs from leaking prematurely in mesoporous carbon carriers, reducing their biological toxicity. Sun et al. [61] achieved reduced DOX leakage through MnO_2_ shell encapsulation of MCNs.

Morphology regulation is an effective method for improving the fundamental physical properties of MCNs. Zhao et al. [62] synthesized MCNs featuring monolayer-arranged spherical mesopores, avoiding the slow mass transfer kinetics caused by multi-layer mesopores and the low accessible surface area of annular mesopores. Recent advancements have introduced asymmetric carbon precursor structures through interfacial self-assembly, microemulsion-confined interfacial assembly, multi-step templating, or phase separation techniques. For example, Peng et al. [63] reported a multifunctional nanoemulsion assembly method to synthesize various dendritic mesoporous nanospheres and multi chamber mesoporous nanospheres. These precursors retain their asymmetric features or wrinkled surfaces after carbonization, which leads to the emergence of new properties or enhances their shape dependent properties. For example, the photothermal conversion efficiency of the asymmetric mesoporous carbon hemisphere integrated with γ-Fe_2_O_3_ and GdPO_4_ nanoparticles designed by Zhang et al. [64] was 1.5 times than that of the corresponding spherical mesoporous carbon. In addition, asymmetric carriers achieve compartmentalized functionality through regional modifications: hydrophobic cavities accommodate chemotherapeutic drugs, surface wrinkles immobilize nucleic acid therapeutics, while hollow structures encapsulate photosensitizers.

### 4.3 High loading capability

The high loading capability of mesoporous carbon-based nanoplatforms makes them ideal candidates for drug delivery systems. The large pore volume and high surface area obviously facilitate the efficient loading of therapeutical agents, such as proteins, small molecule drugs and nucleic acids. Tian et al. [65] introduced a new type of cavity into MCNs, which significantly increased the pore volume, and the carrying capacity of drug molecules on the basis of ensuring that drug molecules were not leaked at random. The property of high drug loading provides the premise for MCNs to realize multi-drug combination therapy. Two or more drugs with no adverse interaction can be loaded together into MCNs with high drug load to achieve synergistic treatment of multiple diseases. Wang et al. [26] loaded the anti-inflammatory drug celecoxib and the anti-cancer drug DOX into a mesoporous carbon carrier, which inhibited inflammation and tumor angiogenesis while ensured biological safety. Furthermore, the tunable morphology and pore size of mesoporous carbon materials enable to optimize drug loading and release kinetics, contributing to targeting and sustainability of drug delivery. Gui et al. [66] prepared a fluorescent hollow mesoporous carbon sphere (HMCS) for tumor treatment with a larger inner cavity, which was beneficial for improving drug loading capacity. By incorporating 10 different drugs into HMCS, it was found that their maximum loading efficiency reached 42.8% ± 2.7%. The high drug loading capacity of mesoporous carbon-based nanoplatforms helps to reduce the frequency of drug delivery, and more importantly, it provides a prerequisite for the high efficiency of nano-drug therapy.

### 4.4 Functional modification

MCNs surfaces can be functionally modified to optimize their performance for drug delivery, therapy, and diagnosis. Due to the abundant functional groups on the surface of MCNs, exogenous functional groups can be easily modified by covalent binding or non-covalent adsorption to improve biocompatibility. Li et al. [67] proved that polyvinyl pyrrolidone (PVP) could bind to mesoporous carbon surfaces and showed good immunological biocompatibility, increasing their cycle time in the body. In terms of targeted drug delivery, MCNs can achieve tumor-specific delivery by modifying targeting ligands [68]. The drug is released within the target cell, thereby enhancing the therapeutic effect and reducing toxicity to normal cells. Fang et al. [56] used the property that HA could target CD44 receptors overexpressed by tumor cells to modify it on hollow MCNs. The nanoplatform could effectively target tumor cells and achieve their accumulation at the tumor site. The surface of mesoporous carbon materials can also integrate a variety of therapeutic and diagnostic molecules to enable versatile biomedical applications. With the combination of fluorescent probes, mesoporous carbon enables real-time imaging to guide drug release [69]. In addition, MCNs can also form complexes with other materials to achieve sensitivity to changes in the concentration of a substance, which can be widely used in biological detection. Yan et al. [70] prepared a novel Co-MOF-74@MC composite by embedding rod-like Co-MOF-74 crystals into MC. The sensor showed high electrochemical performance in the quantitative detection of pyrazinamide and isonicotinyl hydrazide. It can be seen that the functional modification of MCNs can achieve enhanced biocompatibility, targeting, imaging and biological detection, and greatly expand their application in the field of biomedicine.

### 4.5 Broadband UV–Vis–NIR absorption

MCNs possess ordered pore channels (2–50 nm) and exceptionally high specific surface areas (typically ranging from hundreds to thousands of m^2^/g). The porous structure induces multiple reflections and scattering of incident light within the channels, effectively prolonging the light path and enhancing interaction probability between the material and ultraviolet radiation. Conjugated π-bonds formed by sp^2^-hybridized carbon (such as graphene-like lamellae) exhibit strong absorption in the ultraviolet region (200–400 nm) through π-electron transitions. Amorphous sp^3^-hybridized carbon and oxygen-containing functional groups (e.g., carboxyl and carbonyl) introduced during high-temperature treatment may create localized states, reducing transition energy barriers and extending light absorption to longer wavelengths. The ability to absorb a broad range of ultraviolet and visible light wavelengths enables these nanoplatforms to be utilized in various biomedical technologies, including PTT, imaging, and sensing.

MCNs exhibit good photothermal conversion performance with wide UV–Vis–NIR absorption, thereby having potential for PTT. Furthermore, the wide light absorption of MCNs solves phototoxicity problems. The phototoxicity of PDT limits its application to a great extent. Intravenous patients often need to avoid direct sunlight after treatment until photosensitizer is fully metabolized. Li et al. [29] developed MC-MnO_2_ composites loaded with Ce6. The extensive absorption of MCNs in the NIR spectrum prevent Ce6 from being activated by sunlight, greatly reducing phototoxicity. In addition, MCNs are able to efficiently absorb light at specific wavelengths, and when illuminated light passes through MCNs, they can produce a strong acoustic signal, improving the contrast of photoacoustic (PA) imaging. Due to the excellent light absorption and sound emission characteristics of MCNs, PA imaging can be deeply imaged, suitable for early detection and monitoring of internal lesions such as tumors [71]. Wang et al. [72] loaded DOX and coated targeted peptide TKD on MCNs to improve tumor therapeutic effect by PAI and active targeting. The nanoplatform had significant PAI visualization, which was far superior to monotherapy. In short, the extensive UV–Vis light absorption characteristics of MCNs can effectively avoid phototoxicity, and expand the application of MCNs in PTT, PA imaging and other aspects.

### 4.6 Catalytic capacity

MCNs, known as artificial enzymes, have stable catalytic ability and are not easily affected by external conditions, which can be harnessed for various biomedical applications. The catalytic properties of mesoporous carbon materials enable the degradation of ROS, the conversion of biomolecules, and the modulation of cellular microenvironments. The catalytic efficiency of nanozymes has been applied to numerous biomedical fields and multidimensional therapies, showcasing their potential in precision medicine and drug delivery systems [73]. For example, Lu et al. [74] utilized mesoporous carbon (MC-COOH) nanozymes with GSH oxidase activity and MnO_2_-doped carbon nanozyme to synergistically promote PDT of tumors. Obviously, the introduction of various functional components has enabled MCNs to achieve diversified catalytic functions. By doping inorganic and organic elements in the original MCNs framework, enzyme-like activities can be obtained to enhance the effectiveness of tumor treatment. Su et al. [36] developed single-atom Fe dispersed *N*-doped mesoporous carbon nanospheres (SAFe-NMCNs). After doping N and Fe elements, the MCNs complex obtained catalase-like and POD-like activities, and realized catalytic synergistic therapy with PTT. Moreover, MCNs with various catalytic abilities can be potentially applied to biosensing, bioimaging, and therapeutic interventions, expanding their utility in precision medicine and disease management.

## 5 Applications of mesoporous carbon-based nanoplatforms

Due to their orderly and adjustable pore size structure, large specific surface area and easy surface modification, MCNs and their derivatives are widely used as carriers for biomedical applications such as tumor therapy (e.g., PTT, and PTT-assisted collaborative therapy [[75], [76]]), theranostics (e.g., single/multi-modal imaging guided tumor therapy [77]), delivery of active molecules, antibacterial infection, and biological detection.

### 5.1 Tumor therapy

As a deadly disease, cancer is difficult for human beings to conquer in spite of significant progress in treatment. Traditional cancer treatments kill cancer cells while also impair the physiological function of normal cells [78]. To achieve effective tumor treatment, a variety of therapeutic strategies have been developed, such as PTT. PTT has attracted much attention due to its high selectivity, negligible pain, low side effects and powerful anticancer effect [9]. MCNs and their derivatives are widely applied to PTT owing to their unique structure and distinguished photothermal conversion efficiency [35]. Depending on the prominent characteristics of MCNs and their derivatives, great progress has been made in the research of MCNs in tumor combination therapy, including PTT, PTT-chemotherapy, PTT-PDT, PTT-immunotherapy and PTT-chemodynamic therapy (CDT). For example, the heat generated by the photothermal effect can promote the release of chemotherapy drugs from mesoporous carbon carriers, thus enhancing the chemotherapy effect. In addition, the rise of temperature caused by PTT accelerates blood flow and increases oxygen content at the tumor location, enhancing the efficacy of PDT. Similarly, the temperature rise induced by PTT also enhanced the Fenton/CDT reaction. Moreover, PTT also ablates the tumor into fragments, releasing immune-stimulating molecules and antigens, thereby activating anti-tumor immunity. Compared with drug therapy alone, PTT-assisted tumor synergistic therapy produces a stronger therapeutic effect.

#### 5.1.1 PTT

PTT is the process of converting light into heat energy through PTAs under the irradiation of external light sources, causing local high temperature to ablate tumors. PTT, as a novel antitumor therapy, has the characteristics of minimally invasive, high efficiency and little toxic side effects. At present, common PTAs include metal-based materials, carbon-based materials, MOFs, and so on. Carbon-based materials include CDs, graphene, fullerenes, carbon nanotubes and MCNs. Among them, due to their stable photothermal properties and broad-spectrum absorption characteristics, MCNs are an excellent class of PTAs that can effectively convert absorbed light energy into thermal energy for use in PTT. Wang et al. [79] constructed hollow carbon nanospheres (HCSs) for PTT-based cancer therapy. *In vitro* studies displayed that human breast cancer cells MCF-7/ADR, which had taken HCSs, rapidly heated up after NIR light irradiation, causing cell membrane rupture and directly killing cancer cells.

**Fig. 1:**
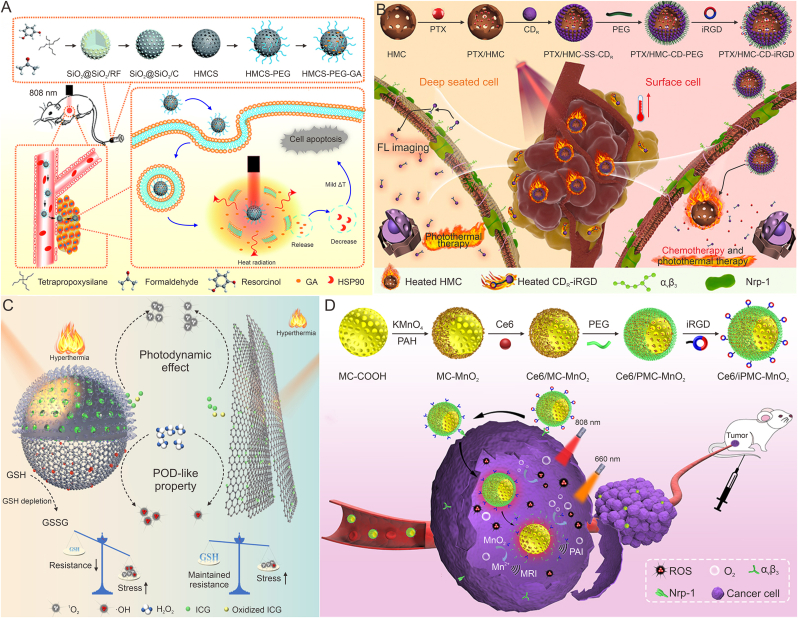
The application of mesoporous carbon nanoparticles (MCNs) in the combined treatment of tumors with photothermal therapy (PTT), PTT-chemotherapy and PTT- photodynamic therapy (PDT). (A) Schematic diagram of the synthesis process of hollow mesoporous carbon nanospheres (HMCS)-polyethylene glycol (PEG)-gambogic acid (GA) nanospheres and photothermal induced tumor cell death. Reproduced with permission from Ref. [75]. (B) The synthesis process of paclitaxel (PTX)/(hollow mesoporous carbon) HMC-(carbon dots) CD-penetrating peptide modification (iRGD) nanospheres and the enhancement of tumor therapy by photothermal chemotherapy in different depths. Reproduced with permission from Ref. [76]. (C) The schematic diagram of the synergistic treatment of indocyanine green (ICG)/mesoporous carbon (MC)-PEG and ICG/graphene oxide (GO)/PEG with combined photothermal and photodynamic effects. Reproduced with permission from Ref. [15]. (D) Preparation of chlorin e6 (Ce6)/mesoporous carbon manganese nanocomposites (iPMC-MnO_2_) nanoplatform and schematic diagram of PTT-PDT synergistic therapy and multiple diagnosis in tumor cells. Reproduced with permission from Ref. [77]. HSP90: heat shock protein 90; CD_R_: red light-emitting carbon dots; FL: fluorescence; GSH: glutathione; POD: peroxidase; GSSG: oxidized glutathione; PAH: polypropylene amine hydrochloride; MRI: magnetic resonance imaging; PAI: photoacoustic imaging; Nrp-1: neuropilin-1; ROS: reactive oxygen species.

When tumor cells are damaged by PTT, they will secrete heat shock proteins (HSPs) to resist thermal damage, which leads to the unsatisfactory efficacy of PTT at low temperature [80]. Hyperthermia PTT can kill tumor cells, but also give rise to destruction to surrounding cells. Therefore, loading HSPs inhibitors through MCNs can enhance the thermal sensitivity of tumor cells, thereby reducing the damage to normal tissues and cells to achieve good PTT effects at lower temperatures [32]. Sun et al. [75] loaded gambogic acid (GA) into hollow mesoporous carbon nanospheres (HMCS) with surface modified 1,2-distearoyl-sn-glycero-3-phosphoethanolamine (DSPE)-polyethylene glycol (PEG) to construct HMCS-PEG-GA NPs. GA had a natural ability to inhibit HSP90, and could specifically target and reduce HSPs in the cytoplasm. Under the 808 nm laser HMCS-PEG-GA group compared with HMCS-PEG group, HSP90 was restrained by about 35% and the HMCS-PEG-GA NPs eliminated almost all cancer cells, showing good therapeutic efficacy under mild PTT (Fig. 1A).

Usually, the direct killing of tumor cells by PTT requires the maintenance of a high temperature at the tumor site. However, while heating up kills tumor cells, it may trigger new blood vessels, which may cause inflammation and tumor metastasis [81]. A large amount of experimental results have indicated that massive inflammation exacerbates tumor growth and metastasis. Therefore, loading of anti-inflammatory drugs through MCNs is thought to solve this problem. Zhang et al. [82] constructed mesoporous carbon/celecoxib@doxorubicin nanoplatform (MCNCD) by modifying the surface of MCN with carboxyl group, grafting celecoxib through the coupling of amino group and carboxyl group, and loading DOX in mesoporous channels. As an anti-inflammatory drug, celecoxib could selectively inhibit cyclooxygenase-2, thereby inhibiting inflammatory response and improving tumor migration. Studies of cell migration using HCT 116 cells revealed that MCNCD almost completely inhibited cancer cell migration.

The photothermal effect induced by PTT can effectively destroy tumor tissue. However, single PTT has insufficient photothermal conversion efficiency, the damage to surrounding tissues, and shallow penetration, leading to inadequate tumor treatment. The association of chemotherapy, PDT, immunotherapy, CDT and PTT can overcome the shortcomings of PTT alone and improve the effect of tumor treatment [24].

#### 5.1.2 PTT-chemotherapy

Chemotherapy is one of the most effective methods for treating cancer. However, chemotherapy drugs have high toxicity and side effects, which not only kill cancer cells but also seriously damage healthy cells. PTT has the advantages of non-invasive and precise treatment, so it is commonly used as an auxiliary treatment of chemotherapy in clinical practice. The local heating induced by PTT sensitizes tumor cells to the action of drugs, thus enhancing the effectiveness of chemotherapy [83]. Furthermore, the treatment of PTT is influenced by tissue depth and laser intensity. High-power laser irradiation will cause damage to human tissues, while chemotherapy can achieve in-depth treatment, so the combination of chemotherapy and phototherapy can achieve excellent tumor treatment effect [84]. Therefore, the synergistic application of MCNs based PTT and chemotherapy provides a new scheme to improve the therapeutic effect of tumor therapy. Tian et al. [66] constructed a hollow mesoporous carbon-coated Fe_3_O_4_ nanoparticle (Fe_3_O_4_@hmC) with a yolk-shell nanostructure of uniform size, and DOX was loaded into the mesoporous carbon channel to form Fe_3_O_4_@hmC-DOX NPs. Compared with free DOX and non-laser irradiation Fe_3_O_4_@hmC-DOX, Fe_3_O_4_@hmC-DOX showed obvious apoptosis of tumor cells under laser irradiation. Consistent with the above reports, Yin et al. [85] prepared polyvinylpyrrolidone (PVP)-modified DOX-loaded mesoporous carbon spheres (HMCS-PVP-DOX). The photothermal interaction initiated by MCNs increased the amount of DOX release and inhibited tumor metastasis and growth.

Different from normal tissues, tumor sites exhibit a special microenvironment of slightly acidic, hypoxic, and high GSH. Therefore, based on the TME, intelligent stimulus-responsive mesoporous carbon-derived nanoplatforms have been proposed. Under the condition of slightly acidic or high GSH concentration in TME, the chemical bond controlling drug release on the mesoporous carbon surface is cut, which realizes drug targeting and controlled release, thereby reducing the toxic side effects of chemotherapy drugs. Zhao et al. [76] constructed a PTX/HMC-CD-iRGD nanoplatform by gating iRGD-modified red light-emitting carbon dots (CD_R_) on a HMC loaded with paclitaxel (PTX) through disulfide bonds. CD_R_ could be used not only as a smart gatekeeper to prevent premature release of PTX, but also a fluorescent agent to visualize the release process. The surface modified iRGD peptide was capable of improving the targeting and penetration ability of PTX/HMC-CD-iRGD. It had been found that under the action of infrared irradiation and high concentration of GSH at the tumor site, PTX/HMC-CD-iRGD nanoplatform was rapidly decomposed into PTX/HMC and CD_R_-iRGD two parts to achieve drug redox/NIR dual-response release, thereby enhancing anti-tumor treatment effect (Fig. 1B). In addition, quantum dots (CDs [76], zinc oxide quantum dots [55]), ultra-small nanoparticles (gold NPs [86]), polymers (polydopamine [87] and polyacrylic acid [88]) and proteins (human serum albumin [89]) have also been attached to MCNs to control drug release. A majority of the drug control and reaction release methods have achieved desired results.

Multidrug resistance is one of the prime causes for the poor effect of chemotherapy. Multidrug resistance arises because cancer cells encode the efflux pump protein P-glycoprotein (P-gp) or over-express multidrug resistance genes (MDR-1) to restraint intracellular drugs from attaining therapeutical standards. Chemosensitizers can down-regulate the expression of efflux proteins. Meanwhile, they enhance the sensitivity of tumor cells to chemotherapeutic drugs [90]. Loading chemosensitizers into MCNs improves the accumulation of chemosensitizers in tumor cells and further improves the therapeutic effect of chemotherapy. Therefore, MCNs-based PTT-chemotherapy therapy can effectively inhibit MDR of cancer cells. Li et al. [91] coated the surface of DOX-loaded MCNs with poly (ethylene glycol)-poly (curcumin-dithiodipropionic acid) (PEG-PCDA) by the thin-film ultrasonic dispersion method to construct the PEG-PCDA/DOX@MCNs nanoparticle drug delivery platform. Curcumin degraded by PCDA at high GSH concentration could down-regulate the expression of drug effector pump and inhibit the activity of ATPase in tumor cells, thereby increasing the accumulation of chemotherapy drugs in cells. In addition, local NIR irradiation can promote cell uptake and inhibit drug efflux, thereby accomplishing effective tumor suppression.

#### 5.1.3 PTT-photodynamic therapy

Due to its potential clinical applications, PDT has received more attention. PDT refers to the local activation of photosensitizers (PSs) under the irradiation of specific wavelengths of light, which produces ROS with strong oxidation capacity in the presence of O_2_, thereby causing damage to tumor tissues. Common PSs include porphyrin, chlorine, phthalocyanine, 5-aminolevulinic acid and other hydrophobic substances. Due to their aggregation and phototoxicity, ROS level generated by PSs in tumor cells is severely limited, and the effect of PDT is also weakened by high hypoxia and GSH in tumor cells [92]. The heat generated by PTT will accelerate the local blood flow speed, increase oxygen delivery as well as alleviate the anoxic environment of the tumor. At the same time, the MCNs rich aperture can effectively load and protect the PSs. Feng et al. [15] used pegylated mesoporous carbon (MC-PEG) as an indocyanine green (ICG) carrier to construct ICG/MC-PEG NPs, which would hinder premature release of ICG, thereby weakening photodegradation and unnecessary phototoxicity. Upon reaching the tumor site, ICG was released from the pores and converted oxygen to ^1^O_2_. Meanwhile, MC-PEG exhibited POD-like ability to deplete GSH. Under the NIR radiation, the ICG/MC-PEG could rapidly be warmed, produce ROS, and further promote oxidative stress through the continuous consumption of GSH, which proved to be more effective than single PTT or PDT in inhibiting tumor (Fig. 1C). MCNs loaded with PSs can reduce the degradation and phototoxicity of PSs, and increase the accumulation in tumor sites. The temperature increase caused by PTT can enhance the absorption of PSs by tumor tissues. Hence, Li et al. [77] constructed the Ce6-loaded mesoporous carbon manganese nanocomposites (iPMC-MnO_2_) with surface-modified PEG and iRGD to enhance Ce6 accumulation at tumor sites. Under NIR irradiation, the heating induced by PTT of MC increased Ce6 uptake by tumor cells. MnO_2_ catalyzed H_2_O_2_ to generate oxygen, which alleviated tumor hypoxia and further enhanced the PDT effect. Compared with single treatment, Ce6/iPMC-MnO_2_ combined with PTT/PDT significantly reduced tumor cell viability, showing a significant anti-tumor effect (Fig. 1D).

In addition to common PSs such as porphyrins, chlorine and phthalocyanine, it has been found that heteroatom doped MCNs can also be used as PSs for PDT. Panda et al. [68] designed a novel drug carrier, N-doped mesoporous carbon (NMCS)-Linker-PEG-polyethylenimine (PEI), using NMCS as the core and PEG-PEI as the shell. Using DCFH-DA as a probe, they proved that NMCS could efficiently produce ROS under NIR laser irradiation by *in vitro* fluorescence assays, thereby inducing cell death through oxidative stress. Therefore, NMCS simultaneously achieve PTT-PDT combined with tumor therapy.

In order to resist internal oxidative stress, tumor cells develop a variety of mechanisms to clear ROS, which leads to the fact that increasing ROS content by PDT alone does not necessarily produce the ideal tumor killing effect. GSH, a vital intracellular antioxidant and ROS scavenger, remains essential for keeping cellular redox balance. The concentration of GSH in tumor cells is about 1,000 times higher than that in normal cells. High level GSH increase ROS clearance from tumor cells and reduce the therapeutic efficacy of ROS-based PDT. In order to reduce the level of GSH in tumor cells, Lu et al. [74] designed a 4T1 membrane coated mesoporous carbon doped with MnO_2_ (CCM) as a photothermal carrier, loaded with photosensitizer Ce6, and constructed a Ce6/CCM nanoplatform. The coating of 4T1 cell membrane with Ce6/CCM NPs achieved efficient tumor homing ability. The excellent GSH depletion ability of MC-Mn was attributed to the fact that MC-COOH exhibited GSH oxidase-like activity and MnO_2_ reacted with GSH to generate GSSH in the acidic environment of the tumor site, thereby achieving dual-GSH depletion. What's more, MnO_2_ could catalyze H_2_O_2_ to produce abundant O_2_, alleviate the hypoxic state of tumor tissues as well as promote ROS generation. This novel biomimetic carbon nanozyme exhibited dual roles as GSH depleter and O_2_ generator, greatly improving the efficacy of PDT.

#### 5.1.4 PTT-immunotherapy

Immunotherapy is known as a cancer treatment that triggers an immune response system in the microenvironment of tumor infection. The high recurrence rate of tumors is a major challenge in the treatment process. PTT combined with immunotherapy is able to effectively prevent tumor recurrence. PTT can induce immunogenic cell death (ICD) and release tumor-associated antigens (TAAs), which are identified by dendritic cells (DC) and presented to T cell receptors in coordination with immune adjuvants. The immune response mediated by activated T cells can even attack metastatic tumor cells, thus limiting tumor recurrence and distant metastasis. As an ideal drug carrier, MCNs can be used to load immune adjuvants, thereby improving the efficiency of antigen-anchored tumors. Song et al. [42] prepared biomimetic NPs using gadolinium-doped mesoporous carbon nanoparticles (Gd-MCNs) as a nanocarium-supported immune adjuvant R837, which were outsourced to tumor extracellular vesicles (EV@Gd-MCNs-R837). Tumor extracellular vesicle encapsulation enabled efficient cellular uptake and targeting of NPs. Tumor ablation and release of TAAs were induced by PTT upon NIR irradiation at 808 nm. At the same time, the release of R837 performed a vaccine-like potency, generating an immune response that attacked the residual tumor cells. Serum levels of cytokine tumor necrosis factor α (TNF-α) and IL-6 were significantly higher in EV@Gd-MCNs-R837 + NIR group than that in the other groups, indicating that PTT and R837 jointly activated the immune response *in vivo*. Studies using a bilateral 4T1 tumor model in BALB/c mice demonstrated that within 21 days, EV@Gd-MCNs-R837 treatment eliminated primary tumors, efficiently suppressed tumor metastasis, and produced long-term immune memory.

In addition to serving as a vector loading immune adjuvant, MCNs can enhance PTT-triggered immunotherapy. The temperature rise generated by PTT can activate the immune system, the initiation and infiltration of T cells, trigger tumor immunogenic responses, induce tumor cell apoptosis, and achieve PTT/immunotherapy synergism to enhance tumor therapy [93]. Wang et al. [53] constructed M27-39@FA-MCNs nanoplatform by modifying a small peptide (M27-39) and FA on MCNs. M27-39@FA-MCNs induced tumor cell apoptosis and restrained tumor growth by targeting the mitochondria of tumor cells, interfering with mitochondrial energy metabolic processes, activating the P53/caspase-3 mitochondrial apoptosis pathway, and reducing the standard of proinflammatory cytokines IL-6, IL-17, interferon-γ (IFN-γ), and IL-1β. Immune checkpoints are protective molecules in the human immune system, restraining inflammatory damage resulted from over-activation of T cells. Tumor cells take advantage of the characteristics of the human immune system by over-expressing immune checkpoint molecules to inhibit the response of the human immune system and escape from human immune surveillance and killing, thereby enhancing the development of tumor tissues [18]. Common immune checkpoint inhibitors are anti-CTLA4 and anti-PD-1 antibodies [94]. By loading immune checkpoint inhibitors on MCNs, the immune checkpoint activity is restrained, the immune response of T cells against tumors is reactivated as well in order to attain anti-tumor effects. Wang et al. [95] constructed the IR792-MCN@ZIF-8-PD-L1 siRNA (IM@ZP) nanoparticle delivery platform by loading IR792 into a channel of MCNs which were coated with zeolitic imidazolate frameworks-8 (ZIF-8), with PD-L1 siRNA being adsorbed on the outer layer. Under NIR laser irradiation, IM@ZP could induce DC maturation and secretion of cytokines TNF-α and IL-6, stimulating the immune response. In the acidic tumor environment, ZIF-8 could release PD-L1 siRNA into the cytoplasm, down-regulate the expression of PD-L1, and improve the effectiveness of cancer immunotherapy.

#### 5.1.5 PTT-chemodynamic therapy

CDT refers to the use of transition metal as a catalyst to trigger Fenton/Fenton-like reaction on the basis of weak acidity of TME and overexpression of H_2_O_2_, catalyzing H_2_O_2_ to produce toxic •OH, thus inducing apoptosis of tumor cells [99]. However, due to poor catalytic conditions in TME, the •OH yield of CDT is not satisfactory. The temperature rise induced by PTT based on MCNs can accelerate Fenton reaction and enhance the effect of CDT treatment. Xu et al. [100] constructed a Cu-doped mesoporous carbon nanosphere loaded with free radical generator 2′-azobis[2-(2-imidazolin-2-yl) propane] -dihydrochloric acid (AIPH) and polyacrylic acid (Cu-MNCS-AIPH@PAA). Cu-MNCS-AIPH@PAA not only had good photothermal properties, but also used terephthalic acid as a probe to detect the yield of •OH. Under 808 nm laser irradiation, the fluorescence intensity of Cu-MNCS-AIPH@PAA group increased significantly with the rise of temperature, indicating that the photothermal effect could significantly increase the •OH yield of CDT. Therefore, it had a good synergistic anticancer effect. Insufficient acidity of the tumor site, low metal ion concentration and low H_2_O_2_ content will reduce the therapeutic effect of CDT. MCNs, loaded with metal ions and combined with PTT to trigger the heating of the tumor site, can accelerate the Fenton reaction, enhance CDT and increase tumor treatment effect. Jiang et al. [101] wrapped Fe/Fe_3_C in a hollow mesoporous graphite-carbon shell and modified the tumor-targeting molecular (PEG-biotin) on the shell surface to prepare FeCNB. The Fe part of Fe/Fe_3_C was Fe^0^, and the Fe_3_C part was Fe^2+^ and Fe^3+^. In TME, FeCNB was corroded by acid to release Fe^2+^, which catalyzed the resolving of H_2_O_2_ to generate •OH. Moreover, Fe^0^ could form a primary cell with graphitic carbon shells under acidic and neutral conditions to generate Fe^2+^ and catalyzed the decomposition of H_2_O_2_ to generate •OH. Similarly, FeCNB could convert NIR light into heat energy, accelerating Fenton reactions, galvanic cell reactions and acid corrosion.

**Fig. 2:**
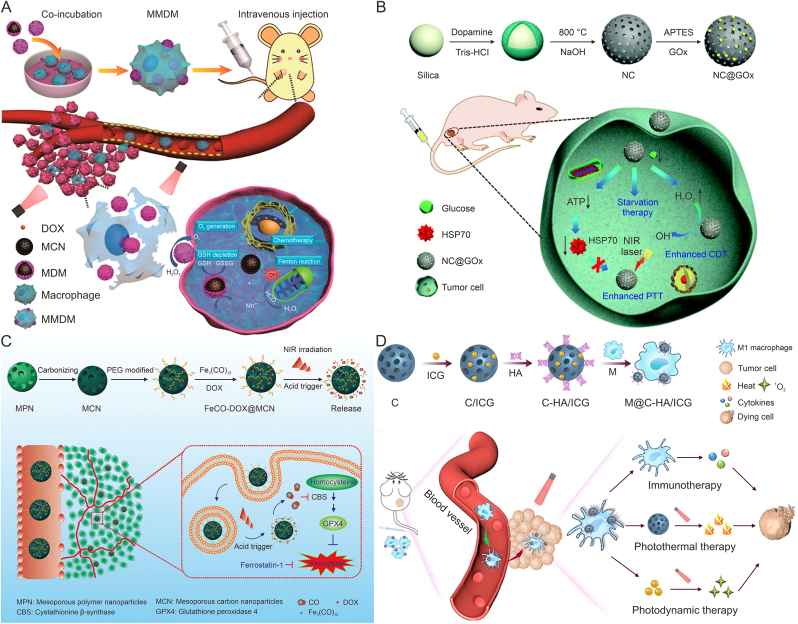
The application of mesoporous carbon nanoparticles (MCNs) in photothermal therapy-chemodynamic therapy (PTT-CDT) and multimodal combined tumor therapy. (A) Schematic representation of construction of the N-doped carbon (NC)@glucose oxidase (GOx) nanoplatform and NC@GOx to achieve PTTCDT-enhanced synergistic tumor therapy by catalytic glucose-triggered starvation therapy, thereby down-regulating heat shock proteins (HSP) expression and enhancing OH production. Reproduced with permission from Ref. [96]. (B) The preparation of Macrophage vehicle internalizing MnO₂-shelled doxorubicin-loaded mesoporous carbon nanosphere (MMDM) and the synergistic therapeutic effect of CDT-PTT generated by MMDM at the tumor site after intravenous injection. Reproduced with permission from Ref. [61]. (C) Schematic diagram of FeCO-doxorubicin (DOX)@mesoporous carbon nanoparticles (MCN) nanoparticle synthesis process and drugs to achieve PTT/chemotherapy/gas therapy combined with anti-tumor therapy. Reproduced with permission from Ref. [97]. (D) Schematic diagram of the synthesis process of macrophage (M)@hollow mesoporous carbon (C)-hyaluronic acid (HA)/indocyanine green (ICG) and PTT/photodynamic therapy/immunotherapy combined with tumor treatment. Reproduced with permission from Ref. [98]. MDM: Manganese dioxide wrapped doxorubicin (DOX)-loaded mesoporous carbon nanosphere; GSH: glutathione; GSSG: oxidized glutathione; APTES: (3-aminopropyl)triethoxysilane; NIR: near-infrared; ATP: adenosine triphosphate; HSP70: heat shock proteins 70; MPN: mesoporous polymer nanoparticles; PEG: polyethylene glycol; CBS: cystathionine β-synthase; GPX4: glutathione peroxidase 4.

Cancer cells have higher H_2_O_2_ levels than normal cells, but endogenous H_2_O_2_ concentrations are still insufficient to achieve satisfactory CDT effects. MCNs loaded with similar POD-like active substances can achieve self-supply of H_2_O_2_, alleviating the insufficient concentration of endogenous H_2_O_2_ in TME. Xu et al. [96] loaded glucose oxidase (GOx) onto N-doped mesoporous carbon nanoparticles (NC) by electrostatic phase absorption to construct NC@GOx NPs. GOx catalyzed glucose into gluconic acid and H_2_O_2_, which not only enhanced the photothermal effect by cutting off the starvation response of nutrients, but also solved the problem of insufficient endogenous H_2_O_2_ content in tumors. Moreover, NC exhibited POD-like activity to decompose H_2_O_2_ to •OH while achieving PTT/CDT enhancement of tumor therapy (Fig. 2A). GSH, as an antioxidant, would eliminate the production of •OH, thereby weakening the effect of CDT. In addition to increasing H_2_O_2_ content in the TME, the reduction of GSH concentration at the tumor site can also enhance CDT effects. Loading GSH depleting agents through MCNs can effectively reduce the concentration of GSH and increase the therapeutic effect of tumor. Sun et al. [61] constructed the macrophage-mediated DOX delivery microsphere (MMDM) vector by encapsulating the MnO_2_ shell into macrophages with DOX-loaded MCNs. After macrophages reaching the tumor site, the infrared light irradiation not only produced the effect of hyperthermia for tumor treatment, but also destroyed the membrane of macrophages. The NPs released and decomposed H_2_O_2_ at the tumor site to generate O_2_, so as to alleviate tumor hypoxia. Meanwhile, GSH reduced MnO_2_ to Mn^2+^, which could mediate Fenton-like reactions to •OH. the CDT effect increased through both GSH depletion and •OH production (Fig. 2B).

#### 5.1.6 PTT-based multiple therapy

Monotherapy is not ideal in the treatment of cancer due to its side effects and low efficiency. In order to improve the treatment effect, multiple treatment modalities such as PTT, chemotherapy, PDT, CDT, and immunotherapy are integrated into one system, and multimodal tumor treatment is used. Multimodal collaborative therapy can combine the merits of multiple single mode therapies to accomplish better tumor therapeutic effect and less side effects. Although the therapeutic effect of bi-modality is better than that of unimodality therapy, multimodal therapy (three or more types based on PTT) further surmounts the disadvantages and enhance the anti-tumor effect.

The combination therapy of CDT and PTT is widely used in tumor therapy, but tumor tissues adjust to a stronger ROS defense system at high ROS level, leading to poor therapeutic efficacy of CDT. When combined PTT/CDT/chemotherapy therapy is used, it can surmount the astriction of PTT penetration deepness as well as reduce drug resistance, improving the sensitivity of tumor tissue to ROS and achieving outstanding therapeutic effects. Xin et al. [102] constructed a DOX/MN-CP-HA nanoplatform by electrostatic adsorption of copper peroxide nanoparticles (CP NPs) onto the surface of DOX-loaded MCNs and coating with HA for PTT/CDT/chemotherapy synergistic tumor therapy. In the TME, CP NPs were decomposed into Cu^2+^ and H_2_O_2_, achieved enhancement in •OH as well as a reduction in GSH, resulting in a strengthened CDT effect. HA modification achieved the targeting function of breast cancer cells. MCNs were loaded with DOX to achieve accelerated release of DOX under photothermal conditions, thereby achieving PTT/CDT/chemotherapy synergistic enhancement of tumor therapy.

Gas therapy based on gases such as carbon monoxide (CO), nitric oxide (NO) and hydrogen sulfide (H_2_S) is considered to be a good adjunct to PTT for cancer treatment [103]. For example, NO and CO have been found to have the ability to enhance tumor damage by therapeutic agents. CO, H_2_S and hydrogen (H_2_) have the effect of reducing inflammation induced by PTT [104]. Therefore, adjunctive gas therapy during PTT may avoid some of the potential side effects present in PTT. Yao et al. [97] loaded DOX and triiron dodecacarbonyl (FeCO) into MCNs to construct FeCO-DOX@MCN nanoplatforms for PTT/chemotherapy/gas therapy combined with anti-tumor therapy. The heating of MCNs under infrared irradiation could produce CO, meanwhile the generated CO could promote the sensitivity of tumor cells to chemotherapy drugs. Compared to treatment alone, the FeCO-DOX@MCN nanoparticles were found to exhibit superior tumor therapeutic effects in MCF-7 tumor mice (Fig. 2C).

In addition, PDT-PTT combined immunotherapy is commonly used as an effective cancer treatment. The inflammation induced by PTT promotes the secretion of chemokines, which further recruits immune cells, so the efficacy of PTT can be enhanced by utilizing the delivery system of immune cells. Macrophages are common immune cells in tumor sites and can be divided into two main subtypes, M1 type and M2 type. M1-macrophages phagocytic tumor cells or secrete cytokines such as NO, ROS, IL-12 and TNF-α to enhance anti-tumor immune response. M2-macrophages can release IL-10, prostaglandin E_2_ (PGE-2), transforming growth factor-beta (TGF-β) and other immunosuppressive factors to enhance tumor development. Studies have shown that carbon-based materials can induce and activate macrophages. Wang et al. [98] constructed M@C-HA/ICG nanosystem by modifying macrophages with hollow mesoporous carbon (C) nanoparticles modified with HA loaded with ICG for combined PDT/PTT/immunotherapy tumor therapy. C nanoparticles promoted the activation of macrophages, while HA with molecular weight <800 kDa transformed macrophages into M1 type. After 808 nm laser irradiation at the tumor site, M@C-HA/ICG obtained good anti-tumor effects through cell membrane destruction, heat-induced apoptosis and pro-inflammatory cytokines (Fig. 2D).

### 5.2 Tumor theranostics

The incidence of tumors is increasing annually, posing a serious threat to health and even life because of their high mortality rate and the lack of effective treatment. Traditional treatment methods include radiotherapy, chemotherapy, etc. They are not proper choices due to the inevitable limitations such as low sensitivity and poor specificity. Besides, multi-drug resistance and cancer recurrence are among the restrictions of the traditional treatment strategies.

With the imaging examination, researchers may see the position and size of the tumor through a variety of crucial bioimaging technologies in the development of tumor theranostics. Many innovative nano-theranostic platforms have evolved into the essential elements of bioimaging technologies, like MRI, fluorescence imaging (FI) and PAI.

#### 5.2.1 MRI guided tumor therapy

MRI is identified as a bioimaging technology that provides the spacial distribution of proton emission signals by the reaction of internal material to the radiation energy of the surrounding environment [74]. It is extremely popular and dependable in the diagnosis and treatment of tumor. Because of the large surface area, three-dimensional pore structure, excellent adsorption, and photothermal properties, MCNs are usually used as MRI contrast agents.

**Fig. 3:**
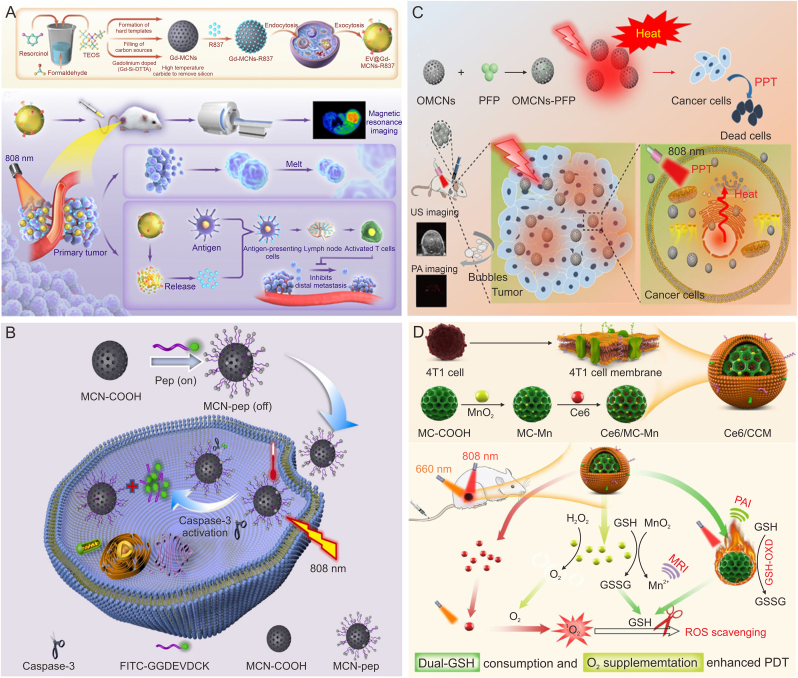
Mesoporous carbon nanoparticles (MCNs) in the field of integrated tumor theranostics. (A) The synthesis process of extracellular vesicle (EV)@Gd-MCNs-Imiquimod (R837) for magnetic resonance imaging (MRI)-guided photothermal therapy (PTT). Reproduced with permission from Ref. [105]. (B) Mesoporous carbon nanospheres (MCN)-based fluorescent nanoprobe (MCN-pep) nanoprobe for caspase3-activated fluorescence tracing and PPT process. Reproduced with permission from Ref. [106]. (C) Oxidized mesoporous carbon nanoparticles (OMCNs) for ultrasound (US) imaging/photoacoustic (PA) imaging guided tumor therapy of PTT. Reproduced with permission from Ref. [107]. (D) The synthesis process of Chlorin e6 (Ce6)/4T1 cell membrane-coated mesoporous carbon nanozyme (CCM) for photoacoustic/magnetic resonance imaging-guided PTT-photodynamic therapy (PDT) combination therapy of tumor. Reproduced with permission from Ref. [74]. TEOS: tetraethyl orthosilicate; FITC: fluorescein isothiocyanate; FITC-GGDEVDCK: FITC-labeled DEVD peptide; PFP: perfluoropentane; GSH: glutathione; GSSG: oxidized glutathione; MC: mesoporous carbon; ROS: reactive oxygen species; PAI: photoacoustic imaging.

MRI contrast agents are split into longitudinal relaxant (T1) and transverse relaxant (T2). The advantage of single-mode (T1 or T2) contrast agents for MRI is that single-mode contrast agents do not have interference-induced signal quenching. Typically, T1-weighted MRI shows higher tissue resolution. Song et al. [105] designed a biomimetic nanoparticle (EV@Gd-MCNs-R837) by modifying Gd-MCNs with the immune adjuvant, R837, and tumor extracellular vesicle coating, with high-definition MRI and superior photothermal conversion efficiency. By plotting the gadolinium concentration curve, the relaxation rate of EV@Gd-MCNs-R837 was determined to be 32.5 mM^−1^s^−1^ Gd, which was much higher than that of Gd-DTPA (4.2 mM^−1^s ^−1^Gd) (Fig. 3A, MCNs can also be used for FI [106] and PAI [107]). In addition, MCN-Gd_2_O_3_ [108] and MCN-Mn_3_O_4_ [109] could also be used as T1-weighted MRI contrast agents. Although T1-weighted MRI has an adequate level of detection, T2 modal MRI is superior in terms of feasibility and is suitable for softer tissues. Tian et al. [66] designed a yolk-shell mesoporous carbon nanocarrier Fe_3_O_4_@hmC. As shown by T2-weighted MRI using a 5.0 T system, Fe_3_O_4_@hmC resulted in stronger longitudinal signals at the tumor site with distinct tumor contours, and quantitative MRI signal analysis of yolk-shelled nanoparticle in the tumor tissue also showed higher results. Other groups have used a similar strategy to develop MCNs-Fe/Fe_3_C [101], and MCNs-Mn_2_O_3_ [110] as T2-weighted MRI contrast agents.

Short T1 blood circulation time and imprecise T2 allow single mode contrast to remain inadequate. The integration of T1-and T2-weighted imaging modalities through dual-mode contrast agents demonstrated substantial potential for enhancing diagnostic precision and consistency in clinical applications [111]. Zhang et al. [112] *in situ* fabricated dual-Fe (g-Fe_2_O_3_ and Fe (PO_3_)_3_) NPs functionalized ordered mesoporous carbon (dual Fe/OMC). From the studies, r1 is estimated to be 9.74 mM^−1^s^−1^, and the r2 value was calculated to be 26.59 mM^−1^s^−1^, which meant that dual-Fe/OMC-24-600 could achieve the effect of T1/T2 dual-modality MRI. Nevertheless, MRI still has the disadvantage of low sensitivity and difficulties in differentiating between benign and malignant tumors.

#### 5.2.2 FI guided tumor therapy

FI technology has the advantages of convenient operation, low cost and good biocompatibility, and is widely used in biomedical imaging of cells, organs, and tissues. This technology offers real-time, high-resolution imaging without the use of radiation, allowing for non-invasive preoperative tumor identification and diagnosis, intraoperative tumor delineation, and postoperative monitoring. However, conventional organic fluorophores are far from satisfactory. In addition to being a fluorescent dye, MCNs can be also used as a fluorescence quencher to construct sensitive quenchers to build sensitive “offline” sensors for multi-diagnostic/targeted imaging of cancer *in vitro* and *in vivo*. Shen et al. [106] developed a fluorescent MCN-pep nanoprobe for real-time monitoring of caspase-3 activity in living cells. MCN acted as a photothermal transducer and fluorescent quencher, inducing apoptosis via PTT under 808 nm NIR and inhibiting fluorescence of fluorescein isothiocyanate (FITC) short peptide length. Then caspase-3 was activated, leading to selective cleavage and degradation of the Asp-Glu-Val-Asp (DEVD) peptide, which resulted in the restoration of FITC fluorescence (Fig. 3B). However, fluorescent nanoprobes relied on external excitation light for diagnostic processes, which cause background fluorescence interference and reduce imaging sensitivity. For instance, Gui et al. [70] designed the fluorescent HMCS. The nanoparticle could spontaneously fluoresce, had long residence times after local injection, and exhibited spherical structures. However, penetration depth issues limit the widespread use of FI *in vivo*.

#### 5.2.3 PAI guided tumor therapy

Due to the high sensitivity, real-time imaging, non-ionizing radiation and optical imagings are mostly used in tumor detection technique. However, the penetration depth of conventional optical imaging is usually very limited, while the currently developed PAI has a higher spatial resolution, deeper penetration depth, and better tissue contrast [74]. Carbon nanomaterials are currently utilized as contrast agents for PAI because of their strong NIR absorption. This enables the local distribution of NPs in tumor regions to be determined [113]. Yao et al. [97] co-loaded hydrophobic iron carbonyl (FeCO) and DOX into MCNs (FeCO-DOX@MCN) to achieve efficient PAI, chemotherapy, and PTT. Based on PAI results, the NIR light energy was efficiently transformed into heat by the FeCO-DOX@MCN nanoplatform, and the resulting CO significantly raised the susceptibility of cells to chemotherapeutics. Furthermore, HMCNs-PEG-GA [75] and HMCNs-DOX [114] could also provide high-resolution PAI.

In addition to carrying drugs and excipients, materials with PAI function, such as gas precursor agents, tend to combine MCNs to achieve PAI signal. For instance, Li et al. [107] combined perfluoropentane (PFP) as a phase-change agent with oxidized mesoporous carbon nanoparticles (OMCNs). PFP-loaded OMCNs nanoplatform irradiated by NIR to produce localized thermotherapy, leading to the rapid phase transition of PFP and generation of air bubbles. The production of bubbles improved the PAI signals, and an excessive local temperature destroyed tumor cells and allowed for photothermal treatment of tumors (Fig. 3C). However, since light transmission in soft tissue is strongly limited by scattering, the imaging depth of PAI can be compromised [115].

#### 5.2.4 Multimodal imaging-guided tumor therapy

Each imaging technology has its unique advantages, however, it comes with certain limitations along the way. For example, FI has a suboptimal depth of penetration, which can be compensated by ultrasound imaging. In this case, researchers tend to integrate the imaging advantages of multiple modalities on the same nanoplatform and develop novel multimodal imaging contrast agents based on this [116]. Multiple imaging techniques provide complementary information to intuitively monitor the metastasis of tumor cells *in vivo* and to accurately measure tumor progression. Dual-modal imaging combines the characteristics of the two imaging technologies and complements with each other, which can better monitor the distribution of therapeutic drugs. Since MCNs are inherently PAI-capable and have adjustable aperture, they can be used to gain insight into the evolution of multimodal imaging techniques that combine PAI, MRI, and other bioimaging techniques. For instance, Lu et al. [74] designed a smart mesoporous carbon nanozyme (Ce6/CCM) with 4T1 membrane-coated and MnO_2_ doped which was used for MRI and PAI. Due to the extensive absorption of carbon-based materials in the NIR region, CCM nanozymes could be used as PAI contrast agents and the prominent T2-weighted MRI signal showed its application in MRI (Fig. 3D).

Although both techniques can provide complementary information, they still may not cover all features of the tumor. Therefore, multiple imaging techniques should be integrated as needed, thus providing more comprehensive understanding of the characteristics of tumors and enhances the accuracy and reliability of diagnosis. Su et al. [36] designed a nitrogen-doped mesoporous carbon nanozymes (SAFe-NMCNs) for photothermal-enhanced ultrasound imaging, FI, and photothermal imaging. SAFe-NMCNs enhanced ultrasound imaging signals by catalyzing the generation of O_2_ from H_2_O_2_ in the tumor microenvironment to provide anatomical information of tumors, which helped to determine the tumor location and size. The distribution of SAFe-NMCNs in the body and enrichment at the tumor site were monitored by FI with high sensitivity. SAFe-NMCNs had strong light absorption in the NIR-II region, and used infrared thermography camera to monitor temperature changes, provide tumor thermograms and enable real-time monitoring of PTT, which provided a new way of thinking about accurate tumor treatment. It is worth noting that multimodal imaging holds tremendous therapeutic promise, but it still faces the drawbacks of high costs and increased workloads.

### 5.3 Antibacterial

Bacterial infection has become a great threat to human health due to its high morbidity and mortality. Antibiotics is a widely used treatment method for the bacterial infectious diseases. Whereas, the abuse of antibiotics has given rise to drug-resistant bacteria and a variety of drug-resistant “superbugs”, which has brought great challenges to clinical treatment. Therefore, it is essential to explore therapies that can efficiently overcome pathogenic bacteria without developing drug resistance. Direct contact between bacteria and MCNs can disrupt the activity of the bacterial wall/membrane, allowing the bacterial contents to leak out and causing the death of the bacteria. In addition, MCNs can generate antibacterial activity against drug-resistant bacteria through PTT due to its excellent photothermal conversion ability [[117], [118]]. MCNs can also be physically or chemically combined with antimicrobials as a carrier of antimicrobials, which can reduce the accumulation of antimicrobials and control their release rate, enhancing the antibacterial effect [[119], [120]].

#### 5.3.1 MCNs as antibacterial agents

**Fig. 4:**
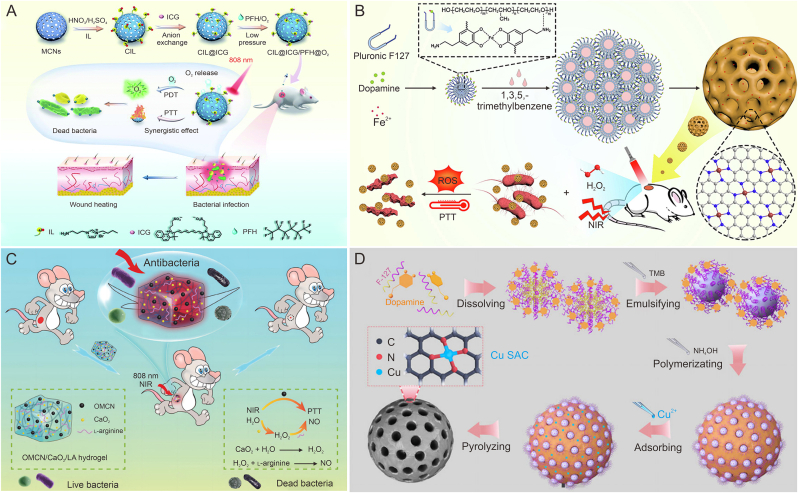
The application of mesoporous carbon nanoparticles (MCNs) in the field of antibacterial. (A) Schematic illustration of the synthesis process of ionic liquids (ILs) was grafted with MCNs (CIL) and CIL@indocyanine green (ICG)/(perfluorohexane)PFH@O_2_ nanoparticles (NPs) and the synergistic antibacterial activity of photothermal therapy/photodynamic therapy (PTT/PDT) produced under near-infrared (NIR) irradiation to promote wound healing. Reproduced with permission from Ref. [117]. (B) Schematic illustration of synthesis of spherical mesoporous Fe–N–C nanozymes and *in vivo* antibacterial principle. Reproduced with permission from Ref. [118]. (C) Schematic diagram of the synthesis process of copper single-atom catalyst (Cu SAC). Reproduced with permission from Ref. [23]. (D) Schematic representation of the synergistic sterilization of oxidized mesoporous carbon nanoparticles (OMCN)/CaO2/L-arginine (LA) hydrogel by NO production and PTT effects. Reproduced with permission from Ref. [128]. ROS: reactive oxygen species; F-127: Pluronic F127; TMB: 3,3′,5,5′-tetramethylbenzidine.

Because of their low toxicity, MCNs have been effectively applied in bio-related research such as biomaterials, drug delivery and antimicrobial agents. MCNs can penetrate the bacterial cell wall with a relatively thin peptidoglycan layer, form intermittent pores in the bacterial cell wall, and induce the decomposition of the bacterial outer membrane through reactive oxygen species, causing phospholipid peroxidation and cell death [121]. In addition, MCNs can adsorb bacteria, destroy the cell membrane and respiratory chain of bacteria, inhibit their energy metabolism, and thus inhibit bacterial division and reproduction. Roy et al. [122] synthesized porous mesoporous carbon nanospheres (MCNSs) with antimicrobial properties. CNSs could destroy the cell membrane of bacteria and cause the gradual degradation of cell morphology. The results showed that the synthesized CNSs had good bactericidal effect against *Escherichia coli* with the lowest bactericidal concentration of 495 ± 0.5 μg/mL. MCNs, due to their excellent photothermal conversion ability, can also kill bacteria through PTT. PTT is considered to be an effective antibacterial method owing to its controllability, non-invasiveness, and few side effects [123]. Local heating caused by PTT could cause cell membrane rupture, protein denaturation and enzyme inactivation, and the formation of drug-resistant bacteria can be effectively inhibited by rapid sterilization at high temperature. However, the therapeutic effect of single PTT therapy is limited. ROS produced by PDT can oxidize damaged bacterial proteins, lipids, and nucleic acids, and can also be used for antibacterial therapy. Therefore, the combination of PTT-PDT can attain better sterilization effect. Zhou et al. [117] constructed the CIL@ICG/PFH@O_2_ nanoplatform by grafting the anionic photosensitizer ICG onto the surface of MCNs loaded with perfluorohexane (PFH) by an ionic liquid group (ILs). The heating caused by MCNs under NIR irradiation promoted the release of oxygen from PFH, which enhanced the PDT effect. Through the synergistic antibacterial action of PTT and PDT, 100 mg/mL^−1^ CIL@ICG/PFH@O_2_ could significantly reduce the survival rate of methicillin-resistant *Staphylococcus aureus* and kanamycin-resistant *Escherichia coli* to less than 1%, showing excellent bactericidal effect and promoting wound healing (Fig. 4A).

MCNs doped with heteroatoms can not only kill bacteria by the heat energy produced by PTT, but also show the activity of nanozymes to enhance the antibacterial effect. Nanozymes with oxidase-like activity or peroxide-like activity could catalyze O_2_ or H_2_O_2_ to produce ROS *in vivo* to destroy bacterial cell membrane and DNA, improve bacterial thermal sensitivity and permeability, and cooperate with PTT photothermal heating to produce physical bactericidal effect, which could be considered as a promising antimicrobial method [124]. MCNs doped with heteroatoms have large specific surface area and abundant pore size, which can increase the contact with the active site, and excellent photothermal properties can accelerate the speed of the catalytic reaction. MCNs can produce good antibacterial effect by combining with PTT. Feng et al. [118] constructed mesoporous Fe–N–C spherical nanozymes for broad-spectrum antimicrobial use. The photothermal conversion efficiency of mesoporous Fe–N–C nanozymes was 23.3%, and the Mi constant K_m_ of H_2_O_2_ was 4.84 mmol/L. And the enzyme-like activity increased gradually with the increase of temperature. In vivo antimicrobial studies showed that mesoporous Fe–N–C could destroy cell membranes and cause bacterial morphology to collapse or split, thus effectively inhibiting bacteria and accelerating wound healing (Fig. 4B).

#### 5.3.2 Deliver antibacterial agents

Antimicrobial therapy through the antibacterial effect produced by MCNs alone is not sufficient to achieve the goal. The effect of sterilization can be improved by loading other antibacterial materials through MCNs. MCNs have attributes of high biocompatibility and easy surface modification, so antibacterial drugs can be combined on MCNs by embedding, adsorption and other ways. Moreover, the large pore and specific surface area of MCNs significantly improve the antibacterial drug loading rate. Moreover, for antibiotics with short half-lives, MCNs loading can provide sustained and controlled drug release, thereby gaining the therapeutic effectiveness of antibiotics and reducing adverse side effects. Nor et al. [125] constructed hollow mesoporous carbon nanospheres (HMCS) loaded with vancomycin (Van) to improve antibiotic bactericidal efficacy and reduce side effects. Due to the hydrophobic attraction, Van achieved high loading and sustained release in HMCS, prolonging the time of effective drug concentration. The HMCS-loaded Van showed good biocompatibility and high inhibitory effect against *Escherichia coli* and Staphylococcus epidermidis. In addition, for drugs with poor solubility, MCNs loading can increase their solubility and dissolution rate, thereby improving their antibacterial efficiency. MCNs not only control the release of drugs, but also effectively reduce the amount of drug degradation through the load of MCNs, thus improving their antibacterial effect. Tebipenem pivoxil (TP) is a prodrug of the broad-spectrum antimicrobial drug tebipenem. TP is easily degraded to its active form tebipenem in the gastrointestinal tract, reducing the antimicrobial activity of the drug. Goscianska et al. [126] constructed a modified ordered mesoporous carbon (OMC) loaded with the β-lactam antibiotic TP. MCNs-loaded TP modified by amino functional groups showed higher dissolution, solving the problem of poor solubility and dissolution of TP. The loading of ordered mesoporous carbon could make TP exhibit strong antibacterial activity at higher concentration.

In addition to traditional antibiotics, some metallic nanomaterials have also been used to treat bacterial infections. Common metal nano-antibacterial agents include silver NPs, gold NPs, zinc NPs, copper NPs, and metal oxide NPs. For example, Cu NPs are widely used as relatively safe and biocompatible antibacterial agents, especially for their significant antibacterial activity against drug-resistant bacteria. Cu NPs can not only damage the cell membrane of bacteria, resulting in membrane rupture, but also catalyze the production of ROS to damage the proteins and nucleic acids of bacteria, causing them to lose the ability of division and proliferation [127]. However, high concentrations of Cu NPs can cause toxic damage to normal organs, which seriously limits their application. As a biocompatible nanomaterial, MCNs and their derivatives can be loaded with Cu NPs to control their release rate and avoid the instant release of a large number of metal ions. Zhao et al. [23] constructed an N-doped mesoporous carbon nanosphere supported with a copper monatomic catalyst (Cu SAC). Cu atom could form Cu-Nx coordination structure with N to control Cu release. The experimental results proved that the prepared NPs had very low toxicity. Cu SAC had a high inhibition rate of 99.75% against bacteria, which killed bacteria without producing MDR, and could inhibit the propagation of bacteria and promote wound healing (Fig. 4C). In recent years, gas antibacterial has also become a research hotspot. As an endogenous gas, NO has broad spectrum antibacterial activity. NO can damage bacterial DNA and inhibit bacterial replication and transcription. Lipid peroxidation, which damages the integrity of cell membranes; Inhibit the enzyme activity in the respiratory chain and destroy metabolism; And through the activation of macrophages, regulate the antibacterial ability of the immune system to achieve multiple mechanisms of antibacterial. Zhang et al. [128] constructed self-activated NO-release hydrogels doped with CaO_2_ NPs, L-arginine (LA), and oxidized MCNs for photothermally-assisted bacterial therapy. H_2_O_2_ produced by the reaction of CaO_2_ NPs with H2O could oxidize LA and release NO continuously. Furthermore, the photothermal effect of NIR light irradiation on OMCNs could further promote the production of NO. Through the synergistic effect of PTT and NO, the inactivation rate of OCL hydrogel against Staphylococcus aureus reached 99% (Fig. 4D). In addition, MCNs-supported silver could also produce good antibacterial effects. Torre et al. [129] constructed ordered MCNs modified with silver NPs to improve antimicrobial properties. Silver doped NPs could effectively reduce the cytotoxicity of silver NPs and showed good biocompatibility. In addition, it was found that silver doped ordered mesoporous carbon had the ability to promote the regeneration of epithelium, blood vessel and tissue.

### 5.4 Biological detection

The properties of MCNs determine their wide application in biological detection. For example, porous structures provide a large number of active sites for detection, and high conductivity makes them a popular material in the electrochemical sensing [130]. In addition, the detection capability of MCNs has been greatly enriched and improved through framework, morphology control and functional modification. Therefore, MCNs and MCNs-based composites have been applied to detect large molecules, small molecules, and some metal ions in various biological samples [[131], [132], [133], [134]].

#### 5.4.1 Detection of nucleic acid

Nucleic acid guides protein synthesis, and the detection of nucleic acid content and sequence is beneficial for disease diagnosis. In DNA fluorescence analysis, MCNs have excellent fluorescence quenching and recovery abilities due to their unique affinity for DNA. Liu et al. [48] labeled DNA with FAM and prepared probes using MCNs as a platform. The prepared probe hybridized with the target DNA and formed a double-stranded structure. Then the nitrogen base blocked the interaction between carbon and nitrogen base through p-p stacking, allowing the probe to separate from the carbon nanomaterial, and fluorescence would be restored simultaneously. By recording fluorescence values, the content of target DNA could be calculated. In addition, the nanoprobe has good selectivity for different nucleotide sequences and can recognize sequences with base mismatches. Liu et al. introduced three mismatched target DNA segments into a nanoprobe solution, recorded and compared the fluorescence recovery rates of the three mismatched target DNA segments with T-DNA, and found that the fluorescence recovery rates of the three base mismatched DNA segments were much lower than that of T-DNA. Therefore, MCNs with single nucleotide recognition ability and good detection performance can serve as a fluorescent probe platform for detecting mutated nucleic acids.

MCNs has a large number of uniformly distributed voids and good electron transfer ability, which lays the foundation for its application in electrochemical sensing. miRNA-21 is a biomarker related to breast cancer. The level of miRNA-21 in serum is often very low, which requires selectivity and sensitivity of detection. Deng et al. [135] constructed an electrochemical sensor combining OMC and graphene oxide to detect miRNA-21 content in serum. The highly conductive OMC played an important role in improving the sensitivity of detection by increasing electron transfer.

#### 5.4.2 Detection of proteins

**Fig. 5:**
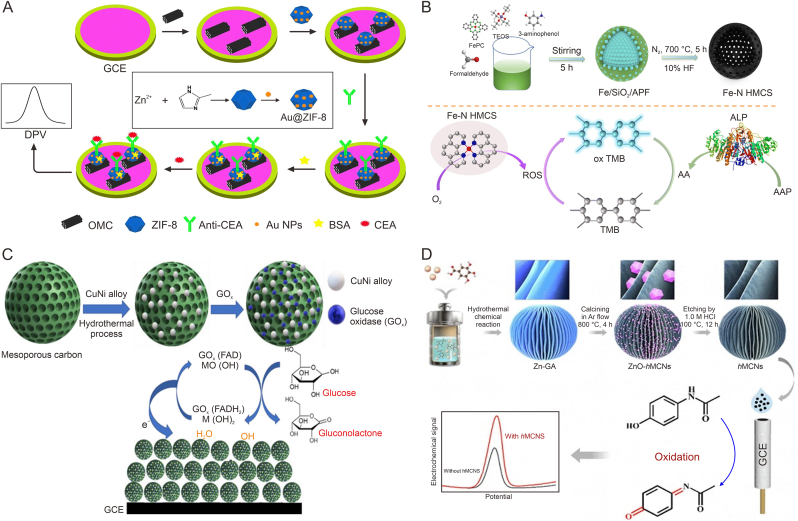
The applications of mesoporous carbon nanoparticles (MCNs) in biological detection. (A) Schematic diagram of preparation of the immunosensors and mechanism of detection of carcinoembryonic antigen (CEA) by immunosensors. Reproduced with permission from Ref. [131]. (B) Schematic diagram of preparation of Fe–N hollow mesoporous carbon spheres (HMCS) and the mechanism of colorimetric analysis of alkaline phosphatase (ALP). Reproduced with permission from Ref. [132]. (C) Schematic diagram of the mechanism for glucose sensing using the CuNi/MCN/glucose oxidase (GOx) catalyst. Reproduced with permission from Ref. [133]. (D) Schematic diagram of the synthesis process of 3D-hierarchical mesoporous carbon nanosheet (*h*MCNS) and the mechanism of electrochemical determination of acetaminophen. Reproduced with permission from Ref. [134]. GCE: glassy carbon electrode; OMC: ordered mesoporous carbon; NPs: nanoparticles; ZIF-8: zeolitic imidazolate frameworks-8; Au@ZIF-8: ZIF-8 modified with Au NPs; BSA: bovine serum albumin; DPV: differential pulse voltammetry; FePC: ironphthalocyanine; TEOS: tetraethyl orthosilicate; APF: 3-aminophenol/formaldehyde; ROS: reactive oxygen species; TMB: 3,3′,5,5′-tetramethylbenzidine; oxTMB: oxidized TMB; AAP: l-ascorbate 2-phosphate; AA: ascorbic acid; FAD: flavin adenine dinucleotide; FADH₂: reduced FAD; Zn-GA: zinc-gallic acid precursor composite.

Proteins are closely related to life activities and protein detection is indispensable for disease diagnosis. Carcinoembryonic antigen (CEA) is a glycoprotein that belongs to a broad-spectrum tumor biomarker and has important clinical value in the differential diagnosis of malignant tumors. OMC has been widely used in sensors due to its excellent properties such as high specific surface area, good conductivity, and high thermal stability. Zhang et al. [131] developed an electrochemical immunosensor based on gold nanoparticle modified zeolite imidazolate framework and OMC for detecting CEA. OMC coated on the electrode surface provided a supporting matrix for the zeolite imidazolate framework and improved the conductivity of the immunosensor (Fig. 5A). Although the electrochemical immunoassay method has good selectivity, its stability is low. Therefore, Hoseynidokht et al. [136] based on the specially prepared mesoporous carbon foam material, used the electrochemical biosensor method to determine the content of CEA in human serum. Conductivity is the most important factor in electrochemical biosensors, and MCNs have high conductivity, high specific surface area, and good biocompatibility, making them widely used in electrochemical biosensors.

In addition, MCNs can be used as carbon nanozymes for colorimetric sensing of alkaline phosphatase (ALP). Rich pore structure and large surface area will facilitate mass transfer processes and expose active sites. Chen et al. [132] constructed a Fe–N hollow mesoporous nanosphere (Fe-N HMCS), which could be regarded as an oxidase with a rich porous structure. Fe-N HMCS oxidizes dissolved oxygen in water to ROS and simultaneously oxidized 3,3', 5,5'-tetramethylbenzidine (TMB) to blue TMB (oxTMB). Ascorbic acid (AA) produced by ALP hydrolysis converted oxTMB to TMB. Therefore, the concentration of ALP was indirectly obtained by detecting changes in oxTMB concentration. The colorimetric sensing system developed through which could sensitively detect the activity of ALP (Fig. 5B).

#### 5.4.3 Detection of blood glucose

The detection of blood glucose concentration can be used to diagnose and monitor diseases with abnormal glucose metabolism such as diabetes. One method of achieving blood glucose detection is to eliminate interference from various components in the blood. Nanozymes have poor selectivity in biological fluids, mainly due to the exposure of active sites on the surface of NPs. It is possible to consider transferring the active site of the enzyme to a nano restricted channel, which can help achieve selective detection of substrates. Benedetti et al. [137] achieved selective detection of glucose in whole blood by using gold NPs coated in conductive mesoporous carbon shells. The isolated mesoporous carbon channels around the gold nanorods were the site for detecting glucose. In addition, the mesoporous carbon coating had conductivity but no oxidation activity towards glucose, which provided a basis for using electrochemical pulses to control the reaction conditions within the nanochannels and detect the potential at which the reaction occurs. This strategy of controlling the reaction environment through nanoscale confinement has great potential for selective detection of various small molecules in biological media.

In addition, some studies have shown that electrochemical analysis of glucose concentration in sweat can accurately reflect blood glucose levels. However, the glucose content in sweat is relatively low, which requires high sensitivity for electrochemical sensors. Therefore, sensitive and stable biosensors exhibit broad application prospects. Radwan et al. [133] developed a glucose biosensor based on NiCu/mesoporous carbon/GOx nanocomposites to indirectly detect blood glucose concentration by detecting sweat glucose concentration. The porous structure of MCNs led to a higher electron transfer rate and the presence of many active sites. By combining MCNs with GOx, the direct transfer of electrons to the electrode surface increased, improving the sensitivity of detection (Fig. 5C).

#### 5.4.4 Detection of drug concentration

The dosage of drugs in the body is closely related to the therapeutic effect. The appropriate blood drug concentration represents excellent therapeutic effect. If the concentration is too low, the therapeutic effect will be poor, and if the concentration is too high, it may even cause poisoning. Therefore, detecting the content of drugs in the body is crucial for monitoring efficacy and optimizing medication regimens. Usually, plasma drug concentration is used to comprehensively reflect the changes in the amount of drugs in the body.

Yan et al. [134] developed a 3D-hierarchical mesoporous carbon nanosheet (hMCNS) microsphere and used it to perform highly sensitive chemical detection of acetaminophen in rat plasma after administration. Interconnected nanosheets promoted electron transfer and improved the conductivity of the sensor. The ultra-high surface area and many open coordination sites of the layered structure could effectively promote the contact between acetaminophen and the sensor. In addition, a pore size of 30 nm contributed to the mass transport and adsorption of acetaminophen. In summary, hMCNS exhibited excellent electrochemical detection performance (Fig. 5D). Apart from the limitations of expensive instrument, the measurement results of this method are consistent with those of high-performance liquid chromatography (HPLC). Meanwhile, compared with carbon nanotubes and graphene, hMCNS does not exhibit agglomeration, its repeatability and reproducibility improved when used in electrochemical sensors. Obviously, the application prospects of this electrochemical sensor in therapeutic drug monitoring are promising.

In addition, functionalized MCNs-based composites have expanded the application of MCNs in detecting drug concentrations. For example, composite materials based on MCNs and graphite oxide can be used to determine DOX in serum [138], and silica/OMC hybrid composite materials can be used to detect ribavirin in human plasma [139].

#### 5.4.5 Detection of inorganic substances in the body

Inorganic substances in the human body also have a significant impact on physical health. For example, high blood lead levels can inhibit the function of the human nerve system and may cause cognitive impairment. Therefore, regular monitoring of blood lead levels in the body is necessary. Various materials, such as carbon nanotubes and metal oxides, have been proved to effectively detect trace levels of lead ions. However, these modifications often require expensive additional materials and complex procedures. In contrast, MCNs have several advantages in electrochemical applications, including ease of electrode modification, cost-effectiveness, large specific surface area, and excellent conductivity. Boselli et al. [140] developed an electrochemical sensor based on a screen printed carbon electrode modified with Nafion and MC for detecting lead in human blood. The required total sample size was small, only a few drops of blood (200 μL) were needed, and the analysis time was short (1 h), which was conducive to regular monitoring of blood lead levels.

The level of hydrogen peroxide in the human body has a significant impact on human health. The existing analytical techniques for determining hydrogen peroxide include electrochemical, colorimetric, fluorescence, and electrochemiluminescence (ECL) method. Among them, the ECL method stands out due to its small sample size, ease of monitoring, and high sensitivity. Wang et al. [141] developed an electrochemiluminescence biosensor based on CdZnSeS QDs and OMC for high-sensitivity detection of hydrogen peroxide in living cells. OMC, as a highly conductive substrate, could reduce the overpotential of ECL systems and amplify the intensity of ECL by binding with quantum dots, thereby achieving effective electron transfer.

### 5.5 Delivery of oral insoluble drugs

In the study of oral drug delivery systems, MCNs have attracted attention for their excellent properties, including high specific surface area, surface that easy to modify, adjustable particle size, pore size and excellent physical stability. In this section, we focus on the research of MCNs in delivering insoluble drugs for oral administration. The mesoporous structure of MCNs enables drugs to enter into the interior of the carrier in an amorphous state [142]. Additionally, MCNs can effectively increase their specific surface area and contact area between drugs and dissolution media, thus promoting the solubility of insoluble drugs.

The solubility of the drugs is an essential factor that controls the bioavailability of the oral drugs. According to the Biopharmaceutic Classification System (BCS), BCS Class II drugs have good permeability and poor solubility, which limits their oral bioavailability. With drug discovery, about 70% of new drug candidates have shown poor aqueous solubility. Therefore, improving the solubility of BCS class II drugs through pharmaceutical methods is of great significance. Drugs with amorphous state present atomic level structure and the lack of long-range order, which endowed them with high energy state and solubility. Thus, loading drugs into MCN after transforming them into amorphous state can obviously improve the bioavailability of BCS Class II drugs.

Besides, some research showed that MCNs could protect amorphous drugs inside, maintaining their high solubility for an extended period. MCNs can effectively inhibit drug crystallization through their mesopore structure, which may be attributed to the formation of monomolecular drug layer of mesoporous carriers that maintain the physical stability of drugs. In a study by Liu et al. [143], MCNs effectively prevented the generation of drug crystals through its unique nanostructure, while enhancing the solubility and oral bioavailability of water-insoluble drugs. According to our previous research, under long-term experimental conditions of 65% humidity and 25°C, Indomethacin and Celecoxib loaded in mesoporous carbon had a crystallinity below 9% after 12 months of storage [144]. In a word, mesopores allow drug molecules to exist stably in an amorphous or microcrystalline state inside MCNs, thus avoiding decomposition or degradation and ensuring effective release, increasing bioavailability of the loaded drugs.

Moreover, compared with the organic polymer carriers used in traditional solid dispersions, MCNs exhibit excellent physical stability, which makes them more reliable for drug delivery applications. At the same time, the surface of MCNs can be modified or functionalized to enhance its hydrophilicity and bioavailability. As shown in the study by Ye et al. [145], by introducing hydrophilic groups on the surface of MCNs, the dispersion and stability in water could be significantly improved, which further improved the solubility and bioavailability of drugs.

Additionally, studies have shown that the particle size of MCNs markedly affects the oral absorption of nanoparticles. In a study by Ran et al. [146], the size effect of branched polyethyleneimine and polyacrylic acid polymers-functionalized mesoporous carbon (MPP) nanoparticles on enhancing oral absorption of water-insoluble drug was investigated. The results of drug release *in vitro* and pharmacokinetics *in vivo* showed that the size of MPP nanoparticles had a significant effect on oral absorption of fenofibrate. The medium-size (250 nm) MPP nanoparticles had the highest oral bioavailability, which was 1.5-fold compared with the commercial preparation. Furthermore, PEI carbon dots (PCA) functionalization and radioactive isotope were applied to label MPP for tracing the *in vivo* process, and the results indicated that MPP showed easy excretion properties with negligible toxicity.

In addition to improving the water solubility of insoluble drugs and increasing their oral bioavailability, MCNs can also be used for surmounting two barriers of mucus permeation and epithelial absorption, which requires completely opposite surface properties of the nanocarriers for oral administration. Lu et al. [147] reported a strategy that hydrophilic N-(2-hydroxypropyl) methacrylamide copolymer (pHPMA) layer and chitosan modified on the surface of MCNs (HCMCN). As a hydrophilic “mucus-inert” polymer, pHPMA assisted HCMCN to permeate through mucus and shed from the surface of HCMCN during penetration, causing leaking positively charged chitosan and facilitating the epithelial uptake of HCMCN. By promoting mucus permeation and epithelial uptake, the absorption barrier of insoluble drug Probucol was overcome, and its bioavailability increased by 2.76 folds compared to commercial formulations.

In summary, MCNs, known as novel carrier materials for drug delivery, have significant advantages in improving the bioavailability of BCS II drugs. Their unique properties such as high specific surface area, adjustable pore structure, superior physical stability and biocompatibility make MCNs the promising material for drug delivery applications. Future studies will explore the application of MCNs in drug delivery systems, with a view to achieving more efficient and safer drug delivery.

## 6 Conclusion and outlook

Due to the excellent biocompatibility, large specific surface area, and controllable pore structure, MCNs can efficiently load a variety of therapeutic and imaging drugs, achieve targeted drug delivery, showing great potential in improving therapeutic effects and reducing side effects. In this review, the classification, preparation and modification methods of MCNs are comprehensively summarized, with emphasis on the physicochemical properties of MCNs. This article reviews the latest progress of MCNs-based nano-drug delivery systems in the fields of tumor diagnosis and treatment, antibacterial therapy, and biological detection. However, the application of MCNs is faced with challenges.

Although the studies on MCNs have become increasingly mature, there are still some challenges such as translation of MCNs-based nano-delivery systems. Firstly, MCNs are often used to load drugs due to their high porosity and suitable pore size, so precise drug release is the main problem faced by nanocarriers. Chemotherapy drugs or proteins in MCNs should be released at the exact time and place, which not only requires the consideration of tissue penetration, but also the targeting, stimulus response, drug loading and other factors when designing MCNs. Secondly, in order to reduce the side effects of drugs and increase the treatment efficiency, researchers tend to obtain the characteristics of targeting, stimulating response and high penetration to realize the synergistic effect of PTT-based tumor therapy or other therapies through surface modification and doping. However, excessive surface modification and doping can inhibit the nanozymes activity of MCNs, hindering their POD-like activity. Meanwhile, the complex large-scale preparation of multi-component MCNs is also a major limitation for the clinical application of MCNs. Therefore, the balance of theory and practice is also a major difficulty in the widespread application of MCNs. Finally, biosafety should also be considered when theory is transferred to clinical practice owning to the fact that carbon materials are less toxic than metal materials. Some carbon nanomaterials cannot be completely degraded, and their accumulation *in vivo* will cause potential biotoxicity. Some studies have shown that it is feasible to prepare carbon complexes for excretion through the urinary system, but there are still some risks in the complex cellular environment [26,27]. In addition, MCNs-based photothermal effects, cascade catalysis and other therapies put forward higher requirements for MCNs, which may bring potential biotoxicity. Therefore, the biosafety of MCNs-based nano-delivery systems should be fully considered to facilitate the subsequent clinical translation.

More feasible and clinically valuable MCNs-based nanoplatforms need to be developed, and researchers should focus on the following aspects: (1) The biosafety of MCNs *in vivo* should be taken seriously. The process of absorption, distribution, metabolism and excretion of nanomaterials *in vivo* is very complex. More in-depth *in vivo* dynamic behavior research is needed to predict and control their behavior *in vivo* to improve the safety and efficacy of drug delivery. Currently, drugs delivered by MCNs often perform well *in vitro*, but not *in vivo*, especially in abnormal tissues and cells under pathological conditions. Therefore, evaluation of the performance and safety of MCNs *in vivo* is necessary. (2) Design MCNs with diverse functions. Future MCNs should be properly designed to achieve multi-overload drugs based on a full understanding of drug interactions to overcome resistance and provide synergies. Meanwhile, future MCNs should be explored in terms of drug intelligent release (pH, temperature, biomarker concentration, etc.) and the combination of targeting and imaging. By designing the rich functions of the future MCNs, they will have a wide range of applications in the biomedical field and provide a more effective and safer solution for clinical treatment. (3) MCNs should be developed and applied in larger fields, such as antibacterial, wound healing, anti-inflammatory and so on. There is great potential of applying MCNs in the antibacterial field because they can not only kill bacteria, but also load antibacterial drugs and exert PTT to achieve synergistic sterilization. In the future, new antibacterial methods such as MCNs equipped with immunomodulators and gas antibacterial should be developed to provide new strategies for the treatment and prevention of bacterial infection. Wound healing is a complex physiological process involving the synergistic action of a variety of cells, extracellular matrix, and growth factors [148]. As an excellent biological material, mesoporous carbon materials provide an ideal scaffold platform for tissue engineering repair due to their good biocompatibility and adjustable biological activity. Several studies have investigated the application of MCNs to wound healing [129,149]. Their abundant pores provide a good microenvironment for cell adhesion, proliferation, and migration. They can also load various growth factors to accelerate wound healing. Anti-inflammatory research is also an important direction in the study of MCNs. MCNs have been shown to inhibit the secretion of inflammatory molecules through thermal effects. Wei et al. [150] developed a nanomotor that targeted inflammatory macrophages, utilizing MCNs as a platform. Under NIR irradiation, the heat generated by MCNs resulted in the ablation of these inflammatory macrophages. Of course, MCNs can function as transport carriers for anti-inflammatory drugs. For instance, Zhang et al. [82] engineered MCNs loaded with the chemotherapy drug DOX and the anti-inflammatory agent celecoxib; this combination effectively obstructed tumor nutrient supply by inducing intravascular thrombosis while minimizing excessive inflammatory suppression associated with acute tissue necrosis. Looking ahead, there is potential for developing multifunctional anti-inflammatory platforms, such as synergistic MCNs that combine antibacterial and anti-inflammatory properties or those designed to enhance wound healing. However, it is crucial to develop biodegradable MCNs to mitigate their accumulation within tissues. Besides, attention should be given to modifying the surface of MCNs to prevent immune rejection reactions from occurring. It is hoped that MCNs will be applied to more fields to meet the expectations of different patients.

MCNs-based nanodrug delivery systems have shown a broad application prospect in the biomedical field, but there are still some challenges need to be overcome, especially in biosafety and application expansion. Through continuous optimization of material properties, in-depth exploration of biological behavior, breakthrough of large-scale production, and in-depth cooperation with clinical practice are expected to be achieved. We hope that the novel nanodrug delivery system based on mesoporous carbon materials can truly realize a smooth translation from laboratory to clinic, and exert their unique advantages in a wider range of diseases.

## CRediT authorship contribution statement

**Wei Yang:** Validation, Writing – review & editing, Resources, Writing – original draft. **Jinnian Ge:** Writing – review & editing, Investigation, Software, Writing – original draft. **Mohan Jiang:** Writing – original draft, Resources, Validation. **Nan Zhang:** Resources, Writing – review & editing. **Qinghe Yang:** Resources, Investigation. **Kaisheng Nan:** Software, Resources. **Qinfu Zhao:** Writing – review & editing, Conceptualization, Writing – original draft. **Long Wan:** Writing – review & editing, Funding acquisition, Writing – original draft. **Xiaofan Wang:** Writing – review & editing, Supervision, Writing – original draft.

## Declaration of competing interest

The authors declare that there are no conflicts of interest.
